# Association between Polymorphisms in Inflammatory Response-Related Genes and the Susceptibility, Progression and Prognosis of the Diffuse Histological Subtype of Gastric Cancer

**DOI:** 10.3390/genes9120631

**Published:** 2018-12-13

**Authors:** Tatiane K. Furuya, Carlos E. Jacob, Michele T. P. Tomitão, Lizeth C. C. Camacho, Marcus F. K. P. Ramos, José Eluf-Neto, Venâncio A. F. Alves, Bruno Zilberstein, Ivan Cecconello, Ulysses Ribeiro, Roger Chammas

**Affiliations:** 1 Centro de Investigacao Translacional em Oncologia (LIM24), Departamento de Radiologia e Oncologia, Instituto do Cancer do Estado de Sao Paulo (ICESP), Faculdade de Medicina da Universidade de Sao Paulo (FMUSP), Sao Paulo 01246-000, Brazil; michele.tomitao@gmail.com (M.T.P.T.); cordobita01@hotmail.com (L.C.C.C.); rchammas@usp.br (R.C.); 2 Departamento de Gastroenterologia, Hospital das Clinicas da Faculdade de Medicina da Universidade de Sao Paulo (HC-FMUSP), Sao Paulo 01246-000, Brazil; jacob.ce@gmail.com (C.E.J.); brunozilb@gmail.com (B.Z.); icecconello@hotmail.com (I.C.); 3 Departamento de Gastroenterologia, Hospital das Clinicas da Faculdade de Medicina da Universidade de Sao Paulo (HC-FMUSP); Instituto do Cancer do Estado de Sao Paulo (ICESP), Sao Paulo 01246-000, Brazil; marcus.kodama@hc.fm.usp.br (M.F.K.P.R.); ulysses.ribeiro@fm.usp.br (U.R.J.); 4 Laboratorio de Epidemiologia e Imunobiologia (LIM38), Departamento de Medicina Preventiva, Faculdade de Medicina da Universidade de Sao Paulo (FMUSP), Sao Paulo 01246-000, Brazil; jelufnet@usp.br; 5 Departamento de Patologia (LIM14), Hospital das Clinicas da Faculdade de Medicina da Universidade de Sao Paulo (HC-FMUSP), Sao Paulo 01246-000, Brazil; venancio@uol.com.br; 6 CICAP, Anatomia Patologica, Hospital Alemao Oswaldo Cruz, Sao Paulo 01327-001, Brazil

**Keywords:** gastric cancer, diffuse histological subtype of Lauren, genetic polymorphisms, haplotypes, inflammation, genetic association studies, genetic predisposition to disease, disease progression, prognosis

## Abstract

The chronic inflammatory microenvironment and immune cell dysfunction have been described as critical components for gastric tumor initiation and progression. The diffuse subtype is related to poor clinical outcomes, pronounced inflammation, and the worst prognosis. We investigated the association of polymorphisms in inflammatory response-related genes (*COX-2*, *OGG1*, *TNFB*, *TNFA*, *HSPA1L*, *HSPA1B*, *VEGFA*, *IL17F*, *LGALS3*, *PHB*, and *TP53*) with gastric cancer susceptibility, progression and prognosis in a Brazilian sample, focusing on the diffuse subtype. We also performed the analysis regarding the total sample of cases (not stratified for tumor subtypes), allowing the comparison between the findings. We further investigated the polymorphisms in linkage disequilibrium and performed haplotype association analyses. In the case-control study, rs1042522 (*TP53*) was associated with a stronger risk for developing gastric cancer in the sample stratified for diffuse subtype patients when compared to the risk observed for the total cases; CTC haplotype (rs699947/rs833061/rs2010963 *VEGFA*) was associated with risk while rs699947 was associated with protection for gastric malignancy in the total sample. Regarding the associations with the clinicopathological features of gastric cancer, for the diffuse subtype we found that rs699947 and rs833061 (*VEGFA*) were associated with outcomes related to a worse progression while rs5275 (*COX-2*), rs909253 (*TNFB*), and rs2227956 (*HSPA1L*) were associated to a better progression of the disease. In the total sample, rs699947 and rs833061 (*VEGFA*), rs4644 (*LGALS3*), and rs1042522 (*TP53*) were able to predict a worse progression while rs5275 (*COX-2*), rs2227956 (*HSPA1L*), and rs3025039 (*VEGFA*) a better progression. Besides, rs909253 (*TNFB*) predicted protection for the overall and disease-free survivals for gastric cancer. In conclusion, these results helped us to clarify the potential role of these polymorphisms in genes involved in the modulation of the inflammatory response in the pathogenesis of gastric cancer.

## 1. Introduction

Gastric cancer is the fifth most common malignancy and the third leading cause of death due to cancer worldwide [1]. In Brazil, the National Cancer Institute (INCA) estimated around 21,290 new cases for 2018–2019 [2]. This disease has multifactorial etiology and molecular complexity, including *Helicobacter pylori* infection, environmental and host genetic risk factors, which poses some challenges for the discovery of molecular markers [3].

Gastric tumors are mostly adenocarcinomas and present high heterogeneity, which is mainly due to architecture and growth, cell differentiation, histogenesis, and molecular pathogenesis [4]. The most common histopathological classification widely used in clinical practice distinguishes two main subtypes of gastric adenocarcinoma: Diffuse and intestinal subtypes of Lauren [5]. The intestinal subtype forms glandular structures that grow in expansion instead of in an infiltrative pattern and it is usually preceded by a chronic atrophic gastritis [6]. The diffuse subtype is poorly differentiated, characterized by the lack of tumor cellular cohesion in the absence of gland formation [4]. These two main histological subtypes present different carcinogenetic and etiologic pathways, specific mechanisms of communication among tumor and stromal cells, and therefore, they have distinct patterns of growth, apoptosis, angiogenesis, morphogenesis, progression, and metastasis [7]. The incidence of the diffuse subtype has increased and they present a more pronounced desmoplasia and associated inflammation with relative preservation of the overlying mucosa [8]. It usually develops from the chronic inflammation without passing through the intermediate steps of atrophic gastritis or intestinal metaplasia observed in the intestinal subtype [9]. It accounts for approximately 30% of gastric cancer cases and it is related to poor clinical outcomes, the worst prognosis and few treatment options [10]. Due to this, in the present work we focused in the diffuse subtype of gastric cancer.

While there are current attempts to propose a molecular taxonomy for gastric cancers (e.g., tumors positive for Epstein-Barr virus, microsatellite unstable, genomically stable, and chromosomally unstable cancers) [11], the clinical staging based on TNM classification of malignant tumors, together with some other clinicopathological features, such as concurrent inflammation, are still in use for predicting the survival and prognosis of this disease [3,12].

Inflammation has been included in the list of hallmarks of cancer which describes the biological mechanisms acquired during tumor development [13]. Additionally, the chronic inflammatory microenvironment and immune cell dysfunction in the stomach have been described as critical components for both tumor initiation and progression [14]. Our group had previously reported a decrease in the expression of genes related to inflammation and immune response in patients with poor prognosis gastric cancer [15]. Furthermore, host genetic variants, including single nucleotide polymorphisms (SNPs) in inflammatory response-related genes, have also been shown to influence in the inter-individual variation in the modulation of the immune and inflammatory processes and then modulate the stomach mucosal damage and gastric carcinogenesis [16,17].

In this context, host genetic susceptibility, in a complex interaction with environmental factors and life style might modulate the inflammatory response, affecting both the individual susceptibility and the course of this disease. The search for molecular markers which could be used as risk factors or predict the behavior of the tumor together with the conventional methods of diagnosis, staging and prognosis is extremely relevant, especially for the diffuse subtype of gastric cancer as pointed before. Thus, in this study, we aimed to investigate whether polymorphisms in inflammatory response-related genes (*COX-2*, *TNFB*, *TNFA*, *HSPA1L*, *HSPA1B*, *VEGFA*, *IL17F*, *LGALS3*, and *PHB*) and DNA repair genes (*OGG1, TP53*) were associated with risk for the development of the diffuse subtype of the gastric tumors, clinical outcomes and prognosis in a Brazilian sample. We also performed the analysis regarding the total sample of cases (not stratified for tumor subtypes), allowing the comparison between the findings. Additionally, we investigated the polymorphisms in linkage disequilibrium and performed haplotype association analyses.

## 2. Materials and Methods

### 2.1. Study Population

This research is part of a multicenter study which aimed to investigate nine different types of tumors in the Brazilian population [18].

This population-based case-control study was carried out in the Hospital das Clinicas da Faculdade de Medicina da Universidade de Sao Paulo (HC-FMUSP), Sao Paulo-SP, Brazil and all the individuals were recruited between June 2001 and October 2006. The case group (N = 178) was composed of consecutive patients diagnosed with gastric adenocarcinoma who underwent surgical resection. All samples were confirmed histopathologically and were classified according to Lauren’s criteria [5]. The diffuse subtype of tumors comprised 63% of the total sample (N = 112). Tumors were staged following the seventh clinical and pathological TNM staging system proposed by the International Union Against Cancer (UICC)/American Joint Committee on Cancer (AJCC) in 2010 [19]. The exclusion criteria were as follows: Patients with gastric neuroendocrine tumor; previous history of gastric surgery; neoadjuvant therapy before resection (either chemotherapy or radiotherapy); unresectable disease; lesions associated with infectious or inflammatory diseases; patients who did not sign the informed consent; and patients with no biological sample for DNA extraction or with low quality DNA for genotyping. At first, 245 cases were analyzed, but after checking for all the inclusion and exclusion criteria, 178 gastric cancer patients were included in the study.

A comparable group of controls (N = 262) without gastric or other types of cancer was recruited from the same hospital. They presented diseases other than cancer which are not related to risk factors for any of the tumors under investigation in the wide study. Both cases and controls were subjected to standardized interviews, using general questionnaires that included information such as life habits, socio-demographic indicators, self-declared ethnicity, assessment for smoking or alcohol consumption, and detailed cancer family history. All participants or their representatives were communicated about the study protocol and gave us informed consent before they participated in the study. All procedures followed were in accordance with the Helsinki Declaration of 1975 and the research protocol was approved by the Institutional Research Ethics Committee (CAPPesq Number 222/01, 11 July 2001).

Clinicopathological data were obtained from the medical records of all gastric cancer patients. Besides gender and age at diagnosis, other analyzed clinicopathological features included: Lauren’s histological classification, tumor size, perineural, lymphatic and vascular invasion, inflammatory infiltration, desmoplasia, depth of tumor invasion (pT classification), lymph nodes metastasis (based on pN), and distant metastasis (pM) and TNM staging.

All patients were followed from the time of enrollment until March 2016. The study endpoints were overall survival (OS) and disease-free survival (DFS). OS was calculated from the date of pathologic diagnosis/recruitment to the date of the last contact or death. Patients who were still alive at the last contact were considered as a censored event in the analysis. DFS was defined as the time from pathologic diagnosis/recruitment to disease recurrence, metastasis, disease-specific death, or last follow-up.

### 2.2. Polymorphisms Selection

The studied polymorphisms were chosen based on the following criteria: Minor allele frequency (MAF) higher than 5%, described in both 1000 Genome Project and/or Exome Aggregation Consortium (ExAC) databases; SNPs in genes described to be involved in immune and/or inflammatory processes; SNPs previously reported to be associated with cancer risk or cancer outcomes in previews studies in the literature, focusing in gastric cancer; potential functional alterations (quantity or quality) of the final coded product. Finally, we selected 15 SNPs from 11 genes: rs1052133 (*OGG1*); rs909253 (*TNFB*); rs1800629 (*TNFA*); rs2227956 (*HSPA1L*); rs1061581 (*HSPA1B*); rs763780 (*IL17F*); rs4644 (*LGALS3*); rs1042522 (*TP53*); rs699947, rs833061, rs2010963 and rs3025039 (*VEGFA*); rs689466 and rs5275 (*COX-2*); and rs6917 (*PHB*). We also investigated the mutation p.R377H from the *TP53* gene.

### 2.3. Blood Sample Collection and Processing, DNA Extraction and Genotyping Analyses

Blood samples were collected in EDTA and genomic DNA was isolated from peripheral blood lymphocytes using PureLink^®^ Genomic DNA Mini Kit (Invitrogen, Carlsbad, CA, USA), following the procedures described by the manufacturer and stored at -20 °C until use for genotyping.

The following SNPs were genotyped by Polymerase Chain Reaction-Restriction Fragment Length Polymorphism (PCR-RFLP): rs1052133 (*OGG1*); rs909253 (*TNFB*); rs1800629 (*TNFA*); rs2227956 (*HSPA1L*); rs1061581 (*HSPA1B*); rs763780 (*IL17F*); rs4644 (*LGALS3*); and rs1042522 (*TP53*). Primers were designed using Primer-BLAST (https://www.ncbi.nlm.nih.gov/tools/primer-blast/) and/or Primer Quest (https://www.idtdna.com/Primerquest). Secondary structures (hairpins, selfdimers and heterodimers) were checked using Oligo Analyzer (https://www.idtdna.com/calc/analyzer) and homology with other sequences in the genome were analyzed using BLAST (https://blast.ncbi.nlm.nih.gov/Blast.cgi). Restriction enzymes were chosen using NEBcutter v2.0 (http://nc2.neb.com/NEBcutter2/).

PCR was carried out in 25 µL of reaction volume containing 100 ng of genomic DNA using 50 mmol/L of KCl, 10 mmol/L of TrisHCl (pH = 8.5), 1.0 mmol/L of MgCl_2_, 0.4 mmol/L of dNTP, 0.16 µmol/L of each primer and 1 U of recombinant Taq DNA Polymerase (Invitrogen, Carlsbad, CA, USA). The mixture was denaturated for 5 min at 94 °C, passed through 40 cycles of denaturing at 94 °C for 30 s, annealing for 30 s (specific annealing temperatures were described in Table S1) and extension at 72 °C for 30 s, with a final extension at 72 °C for 10 min. RFLP reactions were performed using specific enzymes by New England Biolabs (NEB, Ipswich, MA, USA), with conditions also described in Table S1. Both reactions were performed in Veriti Thermal Cycler (Applied Biosystems, Foster City, CA, USA).

The remaining SNPs were genotyped by TaqMan^®^ assays with Real-Time PCR method: rs699947, rs833061, rs2010963 and rs3025039 (*VEGFA*); rs689466 and rs5275 (*COX-2*); rs6917 (*PHB*), and p.R377H (*TP53*). Real-Time PCR was carried out in 10 µL of reaction volume containing 10 ng of genomic DNA using a TaqMan Universal Master Mix (Applied Biosystems, Foster City, CA, USA) and specific allelic discrimination TaqMan™ SNP Genotyping Assays (Applied Biosystems, Foster City, CA, USA), described in Table S1. The mixture was denaturated for 10 min at 95 °C, passed through 40 cycles of denaturing at 95 °C for 15 s, annealing for 1 min at 60 °C, and final extension at 60 °C for 3 min. The equipments StepOne Plus or 7500 (Applied Biosystems, Foster City, CA, USA) were used for this reaction/analysis.

### 2.4. Statistical Analyses

The analyses were performed using SPSS^®^ 18.0, considering *p*-value lower than 0.05 as statistically significant. Allele and genotypic frequencies were calculated by allele counting and the Hardy-Weinberg Equilibrium (HWE) for each polymorphism was tested using Χ^2^ test.

All the association analyses were performed under four different genetic models, considering “a” as the less frequent allele: Genotype (AA versus Aa *versus* aa), Dominant (AA *versus* Aa + aa), Recessive (AA + Aa *versus* aa), and Allele/Multiplicative (A *versus* a) Models. Χ^2^ or Fisher’s Exact tests were used to compare the differences in the distribution of categorical variables (gender, ethnicity, educational level, smoking and drinking status, and genotype frequencies) between cases and controls as well as to analyze the associations of the clinicopathological features among genotypes and between histological subtypes. Age was compared between histological subtypes using Mann-Whitney test. Moreover, the association between the polymorphisms and the risk of gastric cancer were estimated by Odds Ratios (ORs) and their 95% Confidence Intervals (95% CI) using binary logistic regression analysis, adjusted for age, gender, smoking and drinking status. Carriers of the wild-type genotype/allele were used as reference in this analysis.

Linkage disequilibrium (LD) among the polymorphisms was measured by D’ using Haploview 4.2 software (https://www.broadinstitute.org/haploview/haploview) [20]. For haplotype analysis, we included only SNPs that followed the HWE and presented D’ ≥ 0.75. Expectation-Maximization algorithm was calculated to estimate haplotype frequencies and haplotypes with frequency less than 1% were excluded from the analysis. PLINK software (http://zzz.bwh.harvard.edu/plink/) was used to associate haplotypes with both susceptibility for gastric cancer and its clinicopathological features in the diagnosed cases [21].

Survival curves (OS and DFS) were estimated according to the Kaplan-Meier method and the statistical differences were analyzed using the log-rank test. We performed the Cox regression model for estimating the Hazard Ratio (HR) and 95% CI values. The multivariate survival analysis was carried out by adding the studied SNPs to all clinicopathological parameters independently associated with the OS and DFS curves to estimate the effect of each genotype on survival in the presence of other covariates.

All the analyses regarding the individual SNPs were performed considering the sample stratified for the diffuse subtype (N = 112) and also in the total sample of cases (N = 178). Regarding the haplotype analysis, we only performed associations considering the total sample, because of the sample size and the lower frequencies of the haplotypes.

We used the Polyphen-2 (Polymorphism Phenotyping v2) [22] and SIFT (Sorting Intolerant From Tolerant) [23] softwares (available in http://genetics.bwh.harvard.edu/pph2/index.shtml and http://sift.jcvi.org/, respectively) for the in silico prediction of the functional effect of the six missense genetic variants (rs1052133 (*OGG1*), rs2227956 (*HSPA1L*), rs763780 (*IL17F*), rs4644 (*LGALS3*), rs1042522 (*TP53*), and p.R337H (*TP53*)) in the final coded product.

Finally, we checked if the statistical power (1-β) achieved for each association analysis was adequate for the sample size we used in this study, both considering the cases stratified for the diffuse subtype and for the total sample. We considered level of significance α of 5% and effect size of 25%. The softwares GPOWER (http://www.gpower.hhu.de/) [24], Lee (http://www.lee.dante.br/index.html) and STATA 11 were used for this analysis.

## 3. Results

### 3.1. Description of the Sample

The general characteristics of the cases (N = 178) and controls (N = 262) groups are summarized in Table S2. Of these 178 cases, 112, 59, and seven tumors were pathologically classified as diffuse, intestinal, and mixed subtype, respectively. All the association analyses were carried out considering the sample stratified for the diffuse subtype (comprising 63% of the total sample) and were compared to the results regarding the total sample of cases (N = 178).

There were statistically significant differences in the distribution of gender, smoking, and drinking status when comparing cases and control subjects considering both the patients stratified for the diffuse subtype and the total cases. Most cases were smokers or former smokers and had a history of chronic alcohol abuse. Tobacco and alcohol consumption were independently associated with gastric cancer (*p* < 0.001). We included gender, age, smoking, and drinking status as covariates in the multivariate analyses.

The results will be presented separately for markers of susceptibility (case-control study), progression (association with the anatomopathological characteristics) and prognosis (OS and DFS curves) of gastric cancer, considering both the sample composed only by diffuse subtype cases (N = 112) and the total sample of cases (N = 178).

### 3.2. Hardy Weinberg Equilibrium and Genotype/Allele Frequency Distributions

Results of HWE deviation and the genotypic and allelic frequency distributions of the 16 studied genetic variants are presented in Table 1, together with the detailed description of the polymorphisms and their corresponding genes, including other nomenclatures, genomic coordinate, amino acid change (for missense alterations), genotypes, MAF described in 1000 Genome Project, and ExAC databases.

All the analyzed polymorphisms presented allelic frequencies higher than 5%, with exception of the mutation p.R337H *TP53*, which was not detected in the case and control individuals of our study (Table 1). Genotype frequencies of rs1042522 (*TP53*) were significantly different between cases and controls, while allelic frequencies were different regarding rs699947 *(VEGFA*), rs763780 (*IL17F*), and rs1042522 (*TP53*) polymorphisms (Table 1; *p* < 0.05; χ^2^ test).

Considering the sample including both control and case individuals, with exception of rs5275 (*COX-2*), rs763780 (*IL17F*), and rs1042522 (*TP53*) polymorphism, all the other SNPs followed the HWE. When the subsample of cases was analyzed separately, *COX-2* and *TP53* polymorphisms were found to be in HWE (*p* = 0.31 and 0.71, respectively).

### 3.3. Association of the Individual Polymorphisms with Gastric Cancer Susceptibility

At first, we investigated the associations between all selected SNPs and gastric cancer susceptibility stratified for the diffuse histological subtype (N = 112) under the four proposed genetic models (Genotype, Dominant, Recessive and Allele Models).

We found that the Pro (C) allele of rs1042522 (*TP53*) demonstrated an increase of about threefold the risk for gastric cancer in the Genotype, Dominant and Allele Models, after adjustment for the covariates age, gender, smoking, and drinking status in the multivariate analysis. When analyzing the total sample of cases (N = 178), we observed that this association was maintained, but was weaker (increasing roughly twofold the risk for gastric cancer) (Table 2).

Furthermore, A allele of rs699947 (*VEGFA*) was detected as a protection factor for developing gastric cancer in the Genotype, Dominant and Allele Models (Table 2) only when considering the total sample of cases (N = 178). On the other hand, rs763780 (*IL17F*) polymorphism did not show association after the correction for the confounder variables in the multivariate analysis (*p* > 0.05).

### 3.4. Linkage Disequilibrium and Haplotype Associations with Gastric Cancer Susceptibility

For the haplotype association analyses, we first estimated the pairwise LD among the polymorphisms located on chromosome 6 (*TNFB*, *TNFA*, *HSP1AL*, *HSPA1B,* and *VEGFA* genes) using the Haploview 4.2 software. The haplotype analysis requires the assumption of the HWE. While located on chromosome six, the *IL17F* polymorphism was excluded from this analysis once it was not in HWE in both case and control groups (*p* < 0.01). Additionally, we did not perform haplotype association analyses with the two *COX-2* polymorphisms, because rs5275 was not in HWE in both the entire sample and stratified for the control group (*p* < 0.01). Considering D’ ≥ 0.75, we detected that the following polymorphisms were in LD: rs909253 and rs1800629 (*TNFB* and *TNFA*; D’ = 0.87); rs2227956 and rs1061581 (*HSPA1L* and *HSP1B*; D’ = 0.75) and rs699947, rs833061 and rs2010963 (*VEGFA*; D’ = 0.98). The haplotypes composed by *TNFB/TNFA*, *HSP1AL/HSPA1B* and *VEGFA* genes were named as blocks one to three, respectively (Figure S1). The haplotype association analysis was performed only for the entire sample of case and control individuals.

The haplotype association analyses revealed that CTC haplotype of *VEGFA* gene (block three) was associated with gastric cancer susceptibility (*p* = 0.006; Table S3), being more frequent in case (37.6%) than in control individuals (28.6%). ATG and CCC haplotypes (block three) also showed significant associations (*p* < 0.001), with higher frequency in controls than in cases (8.0 and 5.1%, respectively).

### 3.5. Description of the Clinicopathological Features of Gastric Cancer Patients

Clinicopathological parameters of the gastric cancer patients are summarized in Table S4, both considering the total sample and stratifying by histological subtype. According to Lauren’s histological classification, 62.9% of the patients presented the diffuse subtype (N = 112), while 33.1% presented the intestinal subtype (N = 59), and 3.9% were classified as mixed (N = 7). We found that 47.1% of patients presented tumors > 5 cm; 55.8% presented perineural invasion; 60.5% presented lymphatic invasion and 50.6% presented vascular invasion; moderate to intense desmoplasia and inflammatory infiltration were present in 62.2% and 50.5% of the sample, respectively; 81.5% of the sample presented advanced tumors (pT2-T4); 77.4% presented metastasis to lymph nodes and 14.6% presented distant metastasis (in peritoneum, liver, mesocolon, pancreas, and colon). According to TNM staging, we detected that 69.5% were diagnosed and submitted to surgery in higher stages (III and IV).

When comparing these clinicopathological variables between histological subtypes, we observed that the diffuse subtype presented higher prevalence of perineural invasion (*p* = 0.022), moderate to intense desmoplasia (*p* = 0.003), presence of lymph nodes metastasis (*p* = 0.001), and advanced staging (*p* = 0.008) when compared to intestinal subtype (Table S4). Age at diagnosis was also younger in diffuse in comparison to intestinal subtype patients (median (interquartile range) = 60.5 (21) vs. 66 (17) years old; *p* = 0.017). However, in the multivariate analysis, we found that only perineural invasion maintained the statistical significance (*p* = 0.019; OR (95% CI) = 4.3 (1.3–14.0)).

### 3.6. Associations of Individual SNPs with Clinicopathological Features of Gastric Cancer Patients

Initially, we investigated whether the distribution of the genotypic frequencies (in the four genetic models) differed among the categories of the studied clinicopathological features. In the sequence, we adjusted binary logistic regression to calculate the risk of predicting these variables, in both the sample stratified for the diffuse histological subtype (N = 112) and for the entire sample of cases (N = 178). Table 3 describes the significant association found between the analyzed polymorphisms and the clinicopathological variables of the study.

Regarding the sample stratified for the diffuse subtype of gastric cases (N = 112), we found associations between: rs5275 (*COX-2*) and decreased risk of perineural invasion (Genotype and Dominant Models) and desmoplasia (Genotype, Dominant and Allele Models); rs909253 (*TNFB*) and decreased risk of inflammatory infiltration (Genotype and Dominant Models), lymph nodes metastasis (Allele Model) and distant metastasis (Genotype Model); rs2227956 (*HSPA1L*) and decreased risk of perineural invasion (Dominant and Allele Models); rs699947 (*VEGFA*) and increased risk of vascular invasion (Genotype, Dominant and Allele Models); and rs833061 (*VEGFA*) and increased risk of vascular invasion (Dominant and Allele Models) (Table 3). The following polymorphisms were not associated with any of the studied clinicopathological variables: rs1052133 (*OGG1*), rs1800629 (*TNFA*), rs1061581 (*HSPA1B*), rs2010963 and rs3025039 (*VEGFA*), rs763780 (*IL17F*), rs6917 (*PHB*), and rs1042522 (*TP53*) (*p* > 0.05).

Considering the total cases (N = 178), the following associations were detected: rs699947 (*VEGFA*) and increased risk of vascular invasion (Genotype, Dominant and Allele Models); rs833061 (*VEGFA*) and increased risk of vascular invasion and tumors > 5 cm (Dominant and Allele Models); rs4644 (*LGALS3*) and increased risk for desmoplasia (Genotype and Dominant Models); rs1042522 (*TP53*) and increased risk of diffuse histological subtype of gastric cancer (Genotype, Recessive and Allele Models) and desmoplasia (Genotype and Dominant Models); rs689466 (*COX*-2) and decreased risk of diffuse histological subtype of gastric cancer (Allele Model); rs5275 (*COX*-2) and decreased risk of perineural invasion and depth of invasion (Genotype and Dominant Models); rs2227956 (*HSPA1L*) and decreased risk of perineural invasion (Dominant and Allele Models) and desmoplasia (Genotype, Dominant and Allele Models); and rs3025039 (*VEGFA*) and decreased risk of depth of invasion (Recessive Model) and TNM staging (Genotype and Recessive Models) (Table 3). The following polymorphisms were not associated with any of the studied clinicopathological variables: rs1052133 (*OGG1*), rs909253 (*TNFB*), rs1800629 (*TNFA*), rs1061581 (*HSPA1B*), rs763780 (*IL17F*), rs2010963 (*VEGFA*), and rs6917 (*PHB*) (*p* > 0.05).

In summary, considering the sample stratified for the diffuse subtype, we found that the rs699947 and rs833061 (*VEGFA***)** were associated with outcomes related to a worse progression of the disease. In the total sample of cases, this observation was found for rs699947 and rs833061 (*VEGFA*), rs4644 (*LGALS3*), and rs1042522 (*TP53*) polymorphisms. On the other hand, rs5275 (*COX-2*), rs909253 (*TNFB*), and rs2227956 (*HSPA1L*) were associated with variables of a better progression considering the diffuse subtype of the disease and rs689466 and rs5275 (*COX-2*), rs2227956 (*HSPA1L*), and rs3025039 (*VEGFA*) in the total sample of cases.

### 3.7. Associations of Haplotypes with Clinicopathological Features of Gastric Cancer Patients

For the haplotype association analyses with the clinical outcomes, we first estimated the pairwise LD among the polymorphisms in the subgroup of cases located on chromosome 6 (*TNFB*, *TNFA*, *HSPA1L*, *HSPA1B,* and *VEGFA* genes) using the Haploview 4.2 software. We also analyzed the association between the haplotypes of the polymorphism of *COX-2* gene and the clinical outcomes, once the subsample of cases for both polymorphisms (rs689466 and rs5275) followed the HWE. The haplotype association analysis was performed for the entire sample of case and control individuals.

Considering D’ ≥ 0.75, we detected that the following polymorphisms were in LD in the cases group: rs909253 and rs1800629 (*TNFB* and *TNFA*; D’ = 0.79); rs699947, rs833061, and rs2010963 (*VEGFA*; D’ = 0.97), and rs689466 and rs5275 (*COX-2*; D’ = 1.0). The haplotypes composed by *TNFB/TNFA*, *VEGFA* and *COX-2* genes were named as blocks one to three, respectively (Figure S2). The haplotype association analyses were not performed for the sample stratified by diffuse histological subtype.

The haplotype association analysis revealed that, among the clinicopathological features evaluated, we found significant associations between haplotype GG (Block 1) and the presence of perineural invasion (*p* = 0.044); between ACG (Block 2) and the presence of vascular invasion (*p* = 0.012); and between GT (Block 3) and the intestinal histological subtype (*p* = 0.038) (Table S5). Haplotypes with a frequency less than 1% were excluded from the analysis. We did not find associations between the haplotypes and the other clinicopathological variables (*p* > 0.05).

### 3.8. Association of Individual SNPs with Overall and Disease-Free Survival Curves

The means of the OS and DFS survival times were 82.27 ± 5.84 and 94.64 ± 6.27 months, respectively. The maximum follow-up time was 172.5 months (last follow-up in March 2016). At the time of analysis, 99 (55.6%) of the patients had died and 79 (44.4%) presented relapse or death from the disease. Considering 60 months of follow up, OS and DFS were 42.3 and 50.2%, respectively.

The clinicopathological characteristics and their association with OS and DFS curves for the sample stratified for the diffuse subtype of gastric cancer (N = 112) and for the total cases (N = 178) are summarized in Tables S6 and S7, respectively. The univariate analyses showed that all the anatomopathological variables were able to predict worse survival for both OS and DFS, with exception of inflammatory infiltration and desmoplasia for both OS and DFS and perineural invasion only in the diffuse cases for DFS (Tables S6 and S7). All these variables, independently associated with the survival curves in the univariate analyses, were used for adjustment in the multivariate models that investigated the prognostic value for the studied polymorphisms. The demographic variables were not independently associated with survival (*p* > 0.05), and then were not included in the multivariate models.

We calculated the HR values for each SNP and we detected that the G allele of rs909253 (*TNFB*) was associated with a better prognosis when analyzing for both the OS and DFS, in the multivariate models, after adjustments for the independently associated anatomopathological features (Table 4). These associations were observed for both the cases stratified for the diffuse subtype and the total cases (Table 4).

The His allele (A allele) carriers of the rs4644 (*LGALS3*) showed lower DFS time when compared with Pro allele in the Dominant and Allele Models (Table 4) in the total sample of cases (but not in the sample stratified for the diffuse cases). However, this significance was lost when adjusting for the anatomopathological variables in the multivariate model.

We did not find any other association between OS and DFS and the selected polymorphisms of the study (*p* > 0.05).

### 3.9. In Silico Prediction of the Functional Effects of the Missense SNPs

The in silico prediction of the functional effect of the six missense genetic variants in the final coded product were analyzed by Polyphen-2 (http://genetics.bwh.harvard.edu/pph2/index.shtml) and SIFT (http://sift.jcvi.org/) softwares. All the substitutions were considered as benign or tolerated, with the exception of rs4644 (*LGALS*) and p.R337H (*TP53*), which were predicted as possibly pathogenic or deleterious (Table S8).

### 3.10. Statistical Power Calculations for the Sample Size Used in the Study

We calculated if the observed power (1-β) was adequate for the sample size used, for each one of the analysis we performed in the present study, in all the tested genetic models.

For the case-control study, we considered the significance level of 5% (type I error), the genotype and allele frequencies of the significantly associated polymorphisms of *TP53* and *VEGFA* genes and the adjusted OR values by using the Lee software (http://www.lee.dante.br/index.html). We detected an observed statistical power ranging from 70% to 80%, considering the total controls (N = 262) and the sample of cases stratified for the diffuse subtype (N = 112) or the total sample of cases (N = 178).

Regarding the association with the anatomopathological features, we used the GPower software (http://www.gpower.hhu.de/). Considering the significance level of 5%, effect size of 25%, and specific degrees of freedom (depending on the genetic model tested), we observed a statistical power ranging from 65.5% to 75.4% for the cases stratified for the diffuse subtype and 85.7% to 91.6% for the total sample of cases.

For the survival analyses, we used the STATA11 software. When analyzing the cases stratified for with the diffuse subtype, the observed powers were 84.7% and 75.9% for OS and DFS, respectively. For the total cases, we found 93.2% and 86.9% of power for OS and DFS, respectively

## 4. Discussion

We hypothesized that genetic variants resulting in a more pro-inflammatory response could favor the development of a chronically inflamed microenvironment in the gastric tissue, selecting malignant cells that might be able to escape to the immunosurveillance, promoting tumor initiation. Moreover, they could influence in the phenotype aggressiveness, in the biologic behavior and in the clinical course of the disease, favoring the immune system to a more proinflammatory response.

Of the 178 cases with gastric cancer included in our study, 112, 59, and seven tumors were pathologically classified as diffuse, intestinal and mixed subtype, respectively. It is well-described that the two main histological subtypes present different etiological and carcinogenetic pathways [7]. When analyzing a sample including both subtypes mixed, we are likely to lose important association that could be particular for one specific subtype. Thus, we decided to focus our work in the diffuse subtype of gastric cancer, which has a more pronounced inflammation associated and is also related a worse prognosis and poor clinical outcomes [8,10]. Therefore, we aimed to understand the genetic involvement of some polymorphisms in candidate genes related to immune and inflammatory response with the gastric cancer susceptibility, progression, and prognosis, focusing in the diffuse histological subtype of gastric cancer in a sample of the Brazilian population. We also performed the analysis for the total sample, allowing for the comparison between both results. Sufficient statistical power was observed for all the association results considering the sample stratified for both the diffuse subtype (N = 112) and the total cases (N = 178).

The analyses were performed for all four proposed genetic models (Genotype, Dominant, Recessive and Allele Models), which were complementary models in genetic association analysis, decreasing the chance of false-negative results and allowing us to better explore the influence of any allele of a SNP and its mode of inheritance to the studied phenotype, not missing/losing any important finding [25]. Moreover, we investigated the linkage disequilibrium among the studied SNPs located at sites in the same region of the genome and performed the haplotype association analyses, which might provide more statistical power than individual SNP analyses.

The genotype and allele frequencies of the studied polymorphisms were calculated and the MAF were similar to the ones described in the 1000 Genome Project and ExAC databases (Table 1). The p.R337H (*TP53*) mutation was not detected in the case and control individuals in our sample. It has been associated with different types of tumors and is more prevalent in the South region of Brazil [26,27,28,29], probably because of a founder effect [30,31]. This mutation was not relevant for gastric cancer predisposition for the Brazilian sample evaluated in this study.

Considering the entire sample, we found that all SNPs followed the HWE, with exception of rs5275 (*COX-2*), rs763780 (*IL17F*), and rs1042522 (*TP53*) polymorphisms. However, when stratified for the subsamples of controls and cases, both *COX-2* and *TP53* polymorphisms were found to deviate from the HWE only in the control groups. This departure from HWE in control samples could be caused by genotyping error, assortative mating, selection, population stratification, and/or chance [25]. Once *COX-2* polymorphisms were in HWE in the group of cases, we were able to perform their haplotypes association analyses with the clinicopathological parameters.

The Pro allele of rs1042522 (*TP53*) was significantly associated with an increased risk for developing gastric cancer (Genotype, Dominant and Allele Models), after adjustments for covariates in the multivariate analysis. This risk was stronger in the sample stratified for the diffuse histological subtype (roughly threefold) when comparing with the twofold higher risk observed in the total sample. rs1042522 is one of the most studied polymorphism in *TP53* gene and several meta-analyses have described its relevance in increasing the risk for gastric cancer [32,33,34,35]. This change was predicted as benign/tolerated by our in silico analysis, but the wild-type protein with the Arg variant has been shown to be more efficient in inducing the apoptosis than the Pro variant due to its greater ability to interact with MDM2, facilitating its exportation from the nucleus and translocation to the mitochondria [36]. Arg variant is also able to activate transcription and repress the transformation of primary cells [37]. It is important to highlight that the control group of this SNP was not in HWE and this association should be validated in an independent and increased set of samples. Regarding the anatomopathological features, we observed that Pro allele was able to predict the diffuse subtype of gastric cancer (Genotype, Dominant and Allele Models) in the sample including the total cases. The diffuse subtype was demonstrated to be associated with a worse progression of the disease in our sample (Table S4). Furthermore, we also observed an association between the Pro allele and moderate and intense desmoplasia (Genotype, Dominant and Allele Models). The literature describes that a more reactive stroma is linked to a worse prognosis [38].

The *VEGFA* polymorphisms selected for this study are located in the promoter (rs699947 and rs833061), 5′ untranslated (rs2010963), and 3′ untranslated (rs3025039) regions. We detected that the A allele of rs699947 (*VEGFA*) was associated with a protection for developing gastric cancer in the Genotype, Dominant, and Allele Models when considering the total samples of cases, but not stratified for the diffuse subtype. The other selected *VEGFA* polymorphisms were not associated with susceptibility to gastric cancer. However, reinforcing the idea that haplotype studies are usually more informative than studying individual SNPs, we found that three different haplotypes of *VEGFA* (which include rs699947, rs833061, and rs2010963 polymorphisms) were associated with gastric cancer. CTC haplotype was observed in 32.4% of the sample and was more prevalent in cases than in controls. While the ATG and CCC haplotypes also presented differences in the frequencies between cases and controls, their frequencies were very low (4.7% and 3.2%, respectively) and their associations with the disease are probably not biologically relevant. The associations of these *VEGFA* polymorphisms, alone or in haplotypes need to be further explored once the results in the literature are still inconclusive [39,40,41].

Both rs699947 and rs833061 (*VEGFA*) polymorphisms, located at the promoter region, were associated with an increased risk for vascular invasion in our study. The rs699947 was associated in the Genotype, Dominant, and Allele Models and rs833061 in the Dominant and Allele Models, in both the cases stratified for the diffuse subtype and in the total sample. Once again, the observed OR values were stronger in the diffuse subsample than in the total sample. The rs833061 polymorphism was also associated with bigger tumor size (>5 cm) in Dominant and Allele Models, only in the total samples of cases. *VEGFA* has a well-stablished role in processes such as angiogenesis, participating in signaling pathways that lead to vasodilatation and vascular permeability, proliferation, and growth of endothelial cells, cell migration, and differentiation in mature blood vessels [42]. These SNPs might interfere in the *VEGFA* expression, modulating the formation of vessels and facilitating the dissemination of tumor cells to other regions of the body. The haplotype ACG was also associated with vascular invasion, which is probably because of the presence of the two alleles that were individually associated with this characteristic. This is a common example where polymorphisms in linkage disequilibrium were associated with the same variable, being difficult to predict whether one or both are responsible for the observed association. Functional analyses would be necessary for evaluating each one separately to infer which one might cause this association.

In opposition to the two *VEGFA* polymorphisms discussed above, the TT genotype of the SNP located in the 3′ UTR (rs3025039) was able to predict a protection for the disease progression, decreasing the risk for both depth of invasion (Recessive Model) and TNM staging (Genotype and Recessive Models) only when analyzing the total sample of cases. Previous results showed an association between this polymorphism and decreased *VEGFA* levels [43,44].

*COX-2* is associated with inflammatory processes by mediating the conversion of the arachidonic acid to PGH2 [45]. We chose two polymorphisms for investigation in this study that might have a functional role, affecting gene expression regulation, and inflammatory response level: rs689466 and rs5275 are located in the promoter and 3′ UTR of *COX-2*, respectively. We detected an association of G allele of rs689466 (Allele Model) and the intestinal subtype of Lauren. In the haplotype analysis, GT haplotype (presented in 17.4% of our sample) was also more prevalent in the group of individuals with the intestinal subtype. A previous study showed a higher expression of *COX-2* in the intestinal subtype than in the diffuse one [46]. The transcription factor c-MYB is able to recognize and bind to the site with the wild-type A allele of rs689466 in the promoter region, increasing the transcription levels of this gene [45] and the higher expression of *COX-2* has been previously associated with features related to a worse progression of gastric cancer [47,48]. Moreover, the C allele of rs5275 (*COX-2*) was associated with a protection for desmoplasia (Genotype, Dominant, and Allele Models) and perineural invasion (Genotype and Dominant Models) when the sample was stratified for the diffuse subtype and with a depth of invasion and perineural invasion (Genotype and Dominant Models) in the total samples of cases. This SNP is located in the 3′ UTR of the gene and T allele has been described to create a binding site to miR542-3p, promoting its degradation and decreasing its gene expression [49]. However, our study found that this allele was associated with a better progression of gastric cancer.

Once the cytokine expression dysregulation has been described with a key role in inflammatory processes and in malignant tumors, we aimed to investigate if genes from cytokines family could mediate or be involved in both the predisposition and the clinic course of gastric cancer, which has an important inflammatory component involved [50]. Therefore, we selected rs909253 (*TNFB*) and rs1800629 *TNFA* for investigation, which are genes located in tandem in the same cluster, inside Major Histocompatibility Complex (MHC) class II [51]. Considering the stratified cases for diffuse subtype, we found that G allele of rs909253 was associated with the presence of inflammatory infiltration (Genotype and Dominant Models) and with a protection for both lymph nodes (Allele Model) and distant metastasis (Genotype Model). No association was observed when analyzing the total sample of cases, showing the importance of evaluating the different subtypes separately. Relevant associations can be diluted and lost when subtypes with different etiologies are analyzed together. Besides that, this SNP was also associated with a better prognosis for both the OS and DFS, suggesting that it might promote a better progression and prognosis for gastric cancer. While G allele was not associated with the susceptibility of gastric cancer in our study, a previous report demonstrated that it had a protective effect against the development of this disease, in a dose-dependent manner [52]. Another study showed that the other allele (A) of rs909253 was associated with increased transcript and protein levels [53], which could expose the gastric mucosa to a higher cellular damage and inflammation. We did not find associations regarding *TNFA* polymorphism, but we found that *TNFB* and *TNFA* were in linkage disequilibrium and the GG haplotype (presented in 22.6% of the cases) was associated with perineural invasion.

*LGALS3* has an important role in several immune and inflammatory processes [54,55]. We selected the rs4644 (*LGALS3*) polymorphism which is a missense substitution that alters the cleavage pattern for metalloproteases. The protein containing the His variant (A allele) is preferentially cleaved by MMP2 and MMP9, while the Pro variant is resistant to this cleavage [56]. This substitution demonstrated to be potentially pathogenic and deleterious for the protein function by in silico prediction using Polyphen-2 and SIFT softwares. While A allele has been previously associated with a higher risk for breast cancer [57], we did not find an association with susceptibility to gastric cancer in this study. We detected an association with a stronger grade of desmoplasia in the Genotype and Dominant Models. Furthermore, His allele carriers presented a shorter DFS when analyzing the total sample of cases (Dominant and Allele Models); however, it lost the association in the multivariate analysis. These factors are associated with a worse progression of the disease, corroborating previous findings that demonstrated that A allele was associated with a worse evolution of gastric cancer [56,58].

The HSP70 is the most conserved and well characterized heat shock proteins family, which comprises three genes (*HSPA1A*, *HSPA1B,* and *HSPA1L*) located in the region of MHC class III [59]. The rs2227956 (*HSPA1L*) polymorphism changes a hydrophobic and non-polar amino acid (Met) to a polar and neutral one (Thr) in a peptide binding domain, which could alter the binding specificity and altering the biologic function of the protein [60]. However, our in silico prediction showed that this change does not implicate in function alteration in the product and is tolerated/benign. It has been previously described as a protection against the risk for gastric cancer [61]. While we did not find any association with susceptibility, we detected that C allele was able to predict protection for perineural invasion in both total cases and stratified for diffuse subtype (Dominant and Allele Model) and desmoplasia in the total cases (Genotype, Dominant and Allele Models).

The studied polymorphisms that did not present any relevant association in our study (rs2010963 *VEGFA*; rs763780 *IL17F*; rs1061581 *HSPA1B*; rs1052133 *OGG1*; and rs6917 *PHB*) are presented and discussed in Supplementary Materials Section (Text S1). It is important to highlight that this study has some limitations including the moderate sample size and population stratification. We restricted the study to some genes that we believe that are important for the inflammatory response, but these processes are part of complex signaling pathways regulated for innumerous factors, interactions and communication among several molecules. Therefore, further studies are necessary to confirm and elucidate the exact role underlying the involvement of these polymorphisms in the pathogenesis and progression of gastric cancer.

## 5. Conclusions

In conclusion, our study investigated the association of genetic polymorphisms in gastric cancer risk, progression and prognosis, focusing in the diffuse histological subtype in a sample of the Brazilian population. We obtained some evidences of genetic markers and/or molecular mechanisms related to inflammation that could possibly explain inter-individual differences in the susceptibility to gastric cancer, tumor behavior, phenotype aggressiveness and how they could possibly modify the pathogenesis and the disease progression. More studies are necessary to elucidate their possible role in the inflammation and their functional impact in the pathogenesis of gastric cancer.

## Figures and Tables

**Table 1:** Description of the selected polymorphisms, comparison of their genotype/allele frequencies between cases with gastric cancer (N = 178) and control individuals (N = 262), description of the allelic frequencies available in public database and results of the Hardy-Weinberg Equilibrium.

Polymorphism (*Gene*)	Other Names	Genomic Coordinate	Type	Genotype	Genotype N (%)		χ^2^	*p* ^#^	% MAF		χ^2^	*p* ^§^	% MAF 1000 Genomes	% MAF ExAc	HWE	
					Cases	Controls			Cases	Controls					χ^2^	*p*
rs689466 (*COX-2*)	−1195G > A	chr1:186650751	*upstream*	AA	121 (68.0)	157 (61.1)	2.17	0.34	17.4	21.2	1.91	0.17	21.8	-	0.73	0.39
				AG	52 (29.2)	91 (35.4)										
				GG	5 (2.8)	9 (3.5)										
rs5275 (*COX-2*)	+8437T > C	chr1:186643058	3′-UTR (exon 10)	TT	73 (41.0)	108 (41.7)	2.72	0.26	37.4	39.8	0.52	0.47	40.0	-	12.1	<0.001 *
				TC	77 (43.3)	96 (37.1)										
				CC	28 (15.7)	55 (21.2)										
rs1052133 (*OGG1*)	C1245G or Ser326Cis	chr3:9798773	*missense* (Ser→Cis)	CC	114 (64.0)	146 (61.1)	1.56	0.46	19.9	22.8	0.99	0.32	30.2	27.2	1.07	0.30
				CG	57 9 (32.0)	77 (32.2)										
				GG	7 (3.9)	16 (6.7)										
rs909253 (*TNFB*)	+252G > A	chr6:31540313	1^st^ intron	AA	80 (45.2)	97 (42.5)	0.46	0.80	33.9	34.9	0.08	0.77	39.0	-	0.42	0.52
				AG	74 (41.8)	103 (45.2)										
				GG	23 (13.0)	28 (12.3)										
rs1800629 (*TNFA*)	−308G > A	chr6:31543031	*upstream*	GG	138 (77.5)	172 (74.8)	1.45	0.49	13.2	13.9	0.09	0.77	9.0	16.2	5.27	0.02
				AG	33 (18.5)	52 (22.6)										
				AA	7 (3.9)	6 (2.6)										
rs2227956 (*HSPA1L*)	+2437T > C or Thr493Met	chr6:31778272	*missense* (Met→Thr)	TT	155 (87.1)	200 (84.4)	0.91	0.64	6.7	8.4	0.82	0.36	12.3	13.5	1.12	0.29
				TC	22 (12.4)	34 (14.3)										
				CC	1 (0.6)	3 (1.3)										
rs1061581 (*HSPA1B*)	+1267A > G	chr6:31784586	silent (Gln→Gln)	GG	64 (36.2)	69 (29.5)	2.50	0.29	41.5	47.2	2.65	0.10	36.7^b^	-	2.31	0.13
				AG	79 (44.6)	109 (46.6)										
				AA	34 (19.2)	56 (23.9)										
rs699947 (*VEGFA*)	−2578C > A	chr6:43736389	*upstream*	CC	80 (44.9)	93 (36.2)	3.72	0.16	34.0	40.5	3.75	0.049 *	32.5	-	0.95	0.33
				CA	75 (42.1)	120 (46.7)										
				AA	23 (12.9)	44 (17.1)										
rs833061 (*VEGFA*)	−460T > C or −1498T > C	chr6:43737486	*upstream*	TT	72 (40.4)	91 (36.0)	0.89	0.64	37.4	40.1	0.67	0.41	37.0	-	0.26	0.61
				TC	79 (44.4)	121 (47.8)										
				CC	27(15.2)	41 (16.2)										
rs2010963 (*VEGFA*)	+405G > C or +634G > C	chr6:43738350	5′-UTR	GG	68 (38.4)	114 (43.8)	2.17	0.34	38.4	33.7	2.09	0.15	32.6	-	0.02	0.89
				GC	82 (46.3)	117 (45.0)										
				CC	27 (15.3)	29 (11.2)										
rs3025039 (*VEGFA*)	+936C > T	chr6:43752536	3′-UTR (exon 8)	CC	125 (70.6)	177 (70.0)	4.19	0.12	16.4	15.4	0.15	0.7	13.4	-	0.99	0.32
				CT	46 (26.0)	74 (29.2)										
				TT	6 (3.4)	2 (0.8)										
rs763780 (*IL17F*)	7488A > G/His161Arg	chr6:52101739	*missense* (His→Arg)	AA	153 (86.0)	210 (91.3)	3.78	0.15	8.4	4.8	4.47	0.035 *	9.4	6.7	19.7	<0.001 *
				AG	20 (11.2)	18 (7.8)										
				GG	5 (2.8)	2 (0.9)										
rs4644 (*LGALS3*)	+191A > C or Pro64His	chr14:55604935	*missense* (Pro→His)	CC	89 (50.0)	125 (49.4)	1.36	0.51	29.8	28.7	0.13	0.72	29.3	35.5	0.35	0.55
				AC	72 (40.4)	111 (43.9)										
				AA	17 (9.6)	17 (6.7)										
rs6917 (*PHB*)	1630C > T	chr17:47481543	3′-UTR	CC	125 (70.2)	169 (68.1)	0.64	0.73	16.3	16.9	0.06	0.8	16.7	-	0.41	0.52
				CT	48 (27.0)	74 (29.8)										
				TT	5 (2.8)	5 (2.0)										
rs1042522 (*TP53*)	Arg72Pro	chr17:7579472	*missense* (Arg→Pro)	GG	59 (33.1)	118 (48.8)	13.35	0.001 *	41.9	34.7	4.45	0.035 *	45.7	34.0	8.68	0.003 *
				GC	89 (50.0)	80 (33.1)										
				CC	30 (16.9)	44 (18.2)										
p.R337H (*TP53*) ^a^	c.1010G > A or Arg337His	chr17:7574017	*missense* (Arg→His)	GG	0 (0.0)	0 (0.0)								0.0009		
				GA	0 (0.0)	0 (0.0)	-	-	0	0	-	-	-		-	-
				AA	0 (0.0)	0 (0.0)										

N: Number of individuals; chr: Chromosome; ^a^ genetic variation described as mutation; UTR: Untranslated region; ^#^ Genotype Model; ^§^ Allele Model; MAF: Minor allele frequency; MAF 1000Genomes or ExAc: Minor allele frequency described on 1000 Genomes Project or Exome Aggregation Consortium databases; ^b^ information based on UCSC Genome Browser GRCh37/hg19; HWE: Hardy Weinberg Equilibrium in the total sample (considered *p*-value < 0.01); * *p* < 0.05.

**Table 2:** Results of the significant associations detected between rs1042522 (*TP53*) and rs699947 (*VEGFA*) with gastric cancer (both in the cases stratified for the diffuse histological subtype and in the total sample) and the Odds Ratio calculation after adjustments for the covariates in the multivariate logistic regression model.

Polymorphisms (*Gene*)	Genetic Model	Genotype	Cases N (%)	Controls N (%)	*p*	OR (95% CI) ^a^	*p* ^a^
			Total Sample N = 178	Total Sample N = 262			
**Stratified Analyses for the Diffuse Subtype of Gastric Cancer Patients (N = 112)**							
rs1042522 (*TP53*)	Genotype	GG	32 (28.6)	118 (48.8)	0.001 *	1 (Ref)	
		GC	56 (50.0)	80 (33.1)		3.0 (1.7–5.3)	<0.001 *
		CC	24 (21.4)	44 (18.2)		2.6 (1.3–5.3)	0.008 *
	Dominant	GG	32 (28.6)	118 (48.8)	<0.001 *	1 (Ref)	
		GC + CC	80 (71.4)	124 (51.2)		2.9 (1.7–4.9)	<0.001 *
	Recessive	GG + GC	88 (78.6)	198 (81.8)	0.471	1 (Ref)	
		CC	24 (21.4)	44 (18.2)		1.4 (0.8–2.6)	0.254
	Allele ^#^	G allele	120 (53.6)	316 (65.3)	0.003 *	1 (Ref)	
		C allele	104 (46.4)	168 (34.7)		1.9 (1.3–2.7)	0.001 *
**Total Sample of Gastric Cancer Patients (N = 178)**							
rs1042522 (*TP53*)	Genotype	GG	59 (33.1)	118 (48.8)	0.001 *	1 (Ref)	
		GC	89 (50.0)	80 (33.1)		2.30 (1.4–3.7)	<0.001 *
		CC	30 (16.9)	44 (18.2)		1.58 (0.9–2.9)	0.143
	Dominant	GG	59 (33.1)	118 (48.8)	0.001 *	1 (Ref)	
		GC + CC	119 (66.9)	124 (51.2)		2.07 (1.3–3.2)	0.001 *
	Recessive	GG + GC	148 (83.1)	198 (81.8)	0.724	1 (Ref)	
		CC	30 (16.9)	44 (18.2)		1.02 (0.59–1.8)	0.945
	Allele ^#^	G allele	207 (58.1)	316 (65.3)	0.035 *	1 (Ref)	
		C allele	149 (41.9)	168 (34.7)		1.45 (1.1–2.0)	0.017 *
rs699947 (*VEGFA*)	Genotype	CC	80 (44.9)	93 (36.2)	0.160	1 (Ref)	
		CA	75 (42.1)	120 (46.7)		0.64 (0.4–1.0)	0.053
		AA	23 (12.9)	44 (17.1)		0.51 (0.3–0.9)	0.040 *
	Dominant	CC	80 (44.9)	93 (36.2)	0.067	1 (Ref)	
		CA + AA	98 (55.1)	164 (63.8)		0.61 (0.4–0.9)	0.021 *
	Recessive	CC + AC	155 (87.1)	213 (82.9)	0.233	1 (Ref)	
		AA	23 (12.9)	44 (17.1)		0.65 (0.4–1.2)	0.151
	Allele ^#^	C allele	235 (66.0)	306 (59.5)	0.049 *	1 (Ref)	
		A allele	121 (34.0)	208 (40.5)		0.69 (0.5–0.9)	0.016 *

N: Number of individuals; OR: Odds Ratio; 95% CI: 95% Confidence Interval; Ref: Reference; ^a^ adjusted for age, gender, alcohol, and tobacco consumption; ^#^ The Allele Model represents a multiplicative model, with risk being calculated by multiplying the OR × OR values); * *p* < 0.05.

**Table 3:** Significant associations detected between polymorphisms and anatomopathological features (both in the cases stratified for the diffuse histological subtype and in the total sample).

Anatomopathological Characteristics	Polymorphism (*Gene*)	OR (95% CI)									
		Genotype Model				Dominant Model		Recessive Model		Allele Model	
		Aa/AA	*p*	aa/AA	*p*	Aa + aa/AA	*p*	aa/AA + Aa	*p*	allele /A allele	*p*
**Stratified Analyses for the Diffuse Subtype of Gastric Cancer Patients (N = 112)**											
**Perineural Invasion (Yes/No)**	**rs5275 (*COX-2*)**	0.3 (0.1–0.8)	0.012 *	0.7 (0.2–2.3)	0.535	0.4 (0.2–0.9)	0.031 *	1.3 (0.5–3.7)	0.61	0.7 (0.4–1.2)	0.204
	**rs2227956 (*HSPA1L*)**	0.3 (0.1–1.1)	0.07	-	-	0.3 (0.1–0.9)	0.039 *	-	-	0.3 (0.1–0.8)	0.024 *
**Vascular Invasion (Yes/No)**	**rs699947 (*VEGFA*)**	2.2 (0.9–5.3)	0.065	4.8 (1.2–19.4)	0.029 *	2.7 (1.2–6.0)	0.017 *	3.2 (0.9–12.5)	0.084	2.3 (1.3–4.3)	0.007 *
	**rs833061 (*VEGFA*)**	2.1 (0.9–5.0)	0.091	3.5 (0.9–12.9)	0.058	2.4 (1.1–5.4)	0.037 *	2.4 (0.7–8.1)	0.16	2.0 (1.1–3.6)	0.015 *
**Inflammatory Infiltration (moderate + strong/none + weak)**	**rs909253 (*TNFB*)**	3.2 (1.0–9.7)	0.045 *	2.5 (0.6–10.6)	0.202	3.0 (1.1–8.3)	0.040 *	1.5 (0.4–5.3)	0.58	1.9 (0.9–4.1)	0.069
**Desmoplasia (moderate + strong/none + weak)**	**rs5275 (*COX-2*)**	0.2 (0.1–0.9)	0.047 *	0.2 (0.04–1.1)	0.061	0.2 (0.06–0.9)	0.032 *	0.5 (0.1–1.8)	0.30	0.4 (0.2–0.9)	0.031 *
**Lymph Nodes Metastasis (Yes/No)**	**rs909253 (*TNFB*)**	0.5 (0.2–1.8)	0.314	0.3 (0.06–1.0)	0.055	0.4 (0.14–1.28)	0.125	0.4 (0.11–1.17)	0.09	0.5 (0.2–0.9)	0.038 *
**Distant Metastasis (Yes/No)**	**rs909253 (*TNFB*)**	0.2 (0.04–0.9)	0.039 *	0.8 (0.2–3.0)	0.682	0.4 (0.11–1.07)	0.066	1.2 (0.31–4.85)	0.767	0.5 (0.2–1.1)	0.088
**Total Sample of Gastric Cancer Patients (N = 178)**											
**Histological Subtype (Diffuse/Intestinal)**	**rs689466 (*COX-2*)**	0.55 (1.28–1.10)	0.09	0.28 (0.05–1.75)	0.17	0.52 (0.27–1.01)	0.052	0.34 (0.06–2.09)	0.24	0.56 (0.32–0.98)	0.040 *
	**rs1042522 (*TP53*)**	1.40 (0.70–2.80)	0.34	3.60 (1.2–10.8)	0.022 *	1.70 (0.90–3.30)	0.11	2.90 (1.10–8.20)	0.038 *	1.70 (1.10–2.70)	0.026 *
**Tumor Size (>5 cm/≤5 cm)**	**rs833061 (*VEGFA*)**	1.90 (0.90–3.60)	0.06	2.08 (0.9–5.1)	0.11	1.94 (1.10–3.60)	0.034 *	1.49 (0.7–3.4)	0.35	1.56 (1.00–2.40)	0.043 *
**Perineural Invasion (Yes/No)**	**rs5275 (*COX-2*)**	0.49 (0.25–0.96)	0.040 *	0.62 (0.25–1.50)	0.29	0.52 (0.28–0.98)	0.043 *	0.90 (0.40–2.02)	0.79	0.70 (0.45–1.09)	0.11
	**rs2227956 (*HSPA1L*)**	0.40 (0.16–1.00)	0.06	-	-	0.37 (0.15–0.93)	0.034 *	-	-	0.37 (0.15–0.89)	0.045 *
**Vascular invasion (Yes/No)**	**rs699947 (*VEGFA*)**	1.97 (1.01–3.85)	0.047 *	2.54 (0.95–6.78)	0.06	2.10 (1.12–3.92)	0.020 *	1.85 (0.73–4.69)	0.19	1.77 (1.11–2.81)	0.016 *
	**rs833061 (*VEGFA*)**	1.90 (0.90–3.60)	0.075	2.14 (0.84–5.46)	0.11	1.92 (1.02–3.60)	0.043 *	1.57 (0.66–3.73)	0.31	1.59 (1.01–2.50)	0.045 *
**Desmoplasia (moderate + strong/none + weak)**	**rs2227956 (*HSPA1L*)**	0.23 (0.05–0.93)	0.039 *	-	-	0.23 (0.05–0.93)	0.039 *	-	-	0.24 (0.06–0.97)	0.026 *
	**rs4644 (*LGALS3*)**	2.90 (1.17–7.04)	0.021 *	1.40 (0.35–5.51)	0.64	2.45 (1.08–5.56)	0.033 *	0.91 (0.24–3.43)	0.88	1.70 (0.89–3.20)	0.11
	**rs1042522 (*TP53*)**	2.30 (0.90–5.50)	0.74	3.90 (1.1–14.4)	0.039 *	2.60 (1.10–5.60)	0.029 *	2.50 (0.70–8.10)	0.14	2.00 (1.10–3.60)	0.022 *
**Depth of Invasion (t3 + t4/t1 + t2)**	**rs5275 (*COX-2*)**	0.39 (0.17–0.94)	0.040 *	0.42 (0.14–1.27)	0.13	0.40 (0.18–0.91)	0.030 *	0.73 (0.28–1.87)	0.51	0.60 (0.35–1.01)	0.051
	**rs3025039 (*VEGFA*)**	0.90 (0.38–2.13)	0.81	0.11 (0.02–0.65)	0.18	0.66 (0.30–1.43)	0.29	0.12 (0.02–0.66)	0.015 *	0.54 (0.29–1.02)	0.055
**TNM Staging (III + IV/I + II)**	**rs3025039 (*VEGFA*)**	1.76 (0.77–4.02)	0.015 *	0.09 (0.01–0.80)	0.031 *	1.17 (0.56–2.41)	0.68	0.08 (0.01–0.70)	0.022 *	0.82 (0.45–1.50)	0.52

OR: Odds Ratio; 95% CI: 95% Confidence Interval. All the associations analyses were performed under the four genetic models, considering “a” as the less frequent allele: Genotype (AA vs. Aa vs. aa), Dominant (AA vs. Aa + aa), Recessive (AA + Aa vs. aa) and Allele/Multiplicative (A vs. a) Models. Carriers of the wild-type genotype/allele were used as reference for these analyses and were considered as OR = 1. It was not possible to calculate the OR for some genetic models of the rs2227956 *HSPA1L* polymorphism due to the absence of individuals in one of the categories of the corresponding variables; * *p* < 0.05..

**Table 4:** Gastric cancer overall and disease-free survival according to the significant associated polymorphisms (rs909253 *TNFB* and rs4644 *LGALS3*), in the cases stratified for the diffuse histological subtype and in the total sample.

Polymorphism (*Gene*)	Genetic Model	Genotype/Allele	Overall Survival						Disease-Free Survival					
			Cases N	Events N	Mean	log-Rank *p*	HR (95% CI)	*p* ^a^	Cases N	Events N	Mean	log-Rank *p*	HR (95% CI)	*p* ^a^
**Stratified Analyses for the Diffuse Subtype of Gastric Cancer Patients (N = 112)**														
rs909253 (*TNFB*)	Genotype	AA	52	37	54.1	0.073	1 (Ref)		52	33	58.9	0.017 *	1 (Ref)	
		AG	42	27	71.8		0.8 (0.46–1.4)	0.464	42	19	91.6		0.7 (0.4–1.3)	0.280
		GG	18	10	90.8		0.32 (0.1–0.8)	0.013 *	18	7	105.8		0.3 (0.1–0.8)	0.013 *
	Dominant	AA	52	37	54.1	0.049 *	1 (Ref)		52	33	58.9	0.008 *	1 (Ref)	
		AG + GG	60	37	79.1		0.6 (0.4–1.1)	0.078	60	26	98.7		0.5 (0.3–0.9)	0.041 *
	Recessive	AA + AG	94	64	62.5	0.058	1 (Ref)		94	52	73.9	0.040 *	1 (Ref)	
		GG	18	10	90.8		0.35 (0.1–0.8)	0.018 *	18	7	105.8		0.3 (0.1–0.9)	0.025 *
	Allele	A allele	146	101	59.7	0.012 *	1 (Ref)		146	85	68.8	0.002 *	1 (Ref)	
		G allele	78	47	83.2		0.6 (0.4–0.9)	0.007 *	78	33	102.5		0.7 (0.4–0.9)	0.042 *
**Total Sample of Gastric Cancer Patients (N = 178)**														
rs909253 (*TNFB*)	Genotype	AA	80	49	69.8	0.065	1 (Ref)		80	43	75.2	0.02 *	1 (Ref)	
		AG	74	40	85.8		0.8 (0.5–1.2)	0.231	74	27	107.4		0.6 (0.4–1.1)	0.095
		GG	23	10	101.8		0.3 (0.1–0.6)	0.003 *	23	9	105.5		0.3 (0.1–0.7)	0.010 *
	Dominant	AA	80	49	69.8	0.047 *	1 (Ref)		80	43	75.2	0.006 *	1 (Ref)	
		AG + GG	97	50	91.4		0.6 (0.4–0.9)	0.024 *	97	36	108.9		0.5 (0.3–0.9)	0.012 *
	Recessive	AA + AG	154	89	77.9	0.061	1 (Ref)		154	70	91.3	0.160	1 (Ref)	
		GG	23	10	101.8		0.3 (0.1–0.7)	0.006 *	23	9	105.5		0.3 (0.1–0.9)	0.026 *
	Allele	A allele	234	138	75.5	0.015 *	1 (Ref)		234	113	86.2	0.006 *	1 (Ref)	
		G allele	120	60	94.9		0.6 (0.4–0.8)	0.001 *	120	45	109.8		0.5 (0.4–0.8)	0.002 *
rs4644 (*LGALS3*)	Genotype	CC	89	49	85.4	0.371	1 (Ref)		89	32	109.3	0.058	1 (Ref)	
		AC	72	43	71.5		1.1 (0.7–1.8)	0.707	72	38	77.0		1.6 (0.9–2.7)	0.107
		AA	17	7	94.7		0.8 (0.4–2.0)	0.691	17	9	77.2		1.7 (0.8–4.0)	0.193
	Dominant	CC	89	49	85.4	0.442	1 (Ref)		89	32	109.3	0.017 *	1 (Ref)	
		AC + AA	89	50	76.5		1.0 (0.7–1.6)	0.856	89	47	77.7		1.6 (0.9–2.7)	0.075
	Recessive	CC + AC	161	92	80.4	0.402	1 (Ref)		161	70	95.8	0.510	1 (Ref)	
		AA	17	7	94.7		0.8 (0.4–1.9)	0.621	17	9	77.2		1.4 (0.6–3.1)	0.397
	Allele	C allele	250	141	82.1	0.829	1 (Ref)		50	102	100.6	0.029 *	1 (Ref)	
		A allele	106	57	80.1		0.9 (0.7–1.4)	0.935	106	56	78.2		1.4 (0.9–2.0)	0.076

N: Number of individuals; Mean: Mean survival time in months; HR: Hazard Ratio; 95% CI: 95% Confidence Interval; Ref: Reference; ^a^ adjusted for all the anatomopathological features associated in the univariate analysis; * *p* < 0.05.

## Supplementary Materials

The following are available online at http://www.mdpi.com/2073-4425/9/12/631/s1, Table S1 Genotyping details of the studied polymorphisms; Table S2 General characteristics of the studied sample and comparison of the sociodemographic status, smoking and alcohol consumption between controls and cases (both considering the total sample and the cases stratified for the diffuse histological subtype); Figure S1 Representation of the haplotype blocks whose polymorphisms located on chromosome 6 (*TNFB*, *TNFA*, *HSP1AL*, *HSPA1B*, and *VEGFA* genes) were in linkage disequilibrium (LD) in the total sample of cases (N = 178) and controls (N = 262). Table S3. Polymorphisms in linkage disequilibrium and haplotype association analyses with gastric cancer susceptibility. Table S4. Clinicopathological characteristics of the cases with gastric cancer at the time of diagnosis in the total sample and stratified by Lauren’s histological subtypes and results of the comparison of these parameters between Diffuse and Intestinal subtypes. Figure S2 Representation of the haplotype blocks whose polymorphisms were found in linkage disequilibrium (LD) in the subgroup of cases with gastric cancer. Table S5 Polymorphisms in linkage disequilibrium in the sample of cases (N = 178) and haplotype association analyses with anatomopathological features of gastric cancer patients. Table S6 Overall and Disease-free survival by anatomopathological features, stratified for the cases with the diffuse histological subtype (N = 112). Table S7 Overall and Disease-free survival by anatomopathological features in the total sample of gastric cancer patients (N = 178). Table S8 In silico prediction for the functional effect in the final coded protein for the studied polymorphism that lead to amino acid change. Text S1 Discussion of the polymorphisms that did not present any relevant association in our study.
