# Supplementary material for: Association between Polymorphisms in Inflammatory Response-Related Genes and the Susceptibility, Progression and Prognosis of the Diffuse Histological Subtype of Gastric Cancer
Source: Genes (Basel). 2018 Dec 13;9(12):631. doi: 10.3390/genes9120631 (PMC6315504; doi:10.3390/genes9120631)
Supplement: Supplementary file 1 [file genes-09-00631-s001.pdf]

Table S1. Genotyping details of the studied polymorphisms.

**Polymorphisms genotyped by PCR-RFLP**					
**Polymorphism (Gene)**	***Primer sequences* (*Forward* e *Reverse*)**	**Anneling temperature (ºC)**	**Product size after amplification by PCR (bp)**	**Restriction enzyme (Units used per reaction)**	**Fragment size after digestion (bp)**
**rs1052133 (*OGG1*)**	F5'-AGTGGATTCTCATTGCCTTCG-3'<br>R5'-GGTGCTTGGGGAATTTCTTT-3'	57	251	*Fnu4H*I (1U)	251, 155, 96
**rs909253 (*TNFB*)**	F5'-TCTGACTCTCCATCTGTCAGTCTC-3'<br>R5'-GAAGAGACGTTCAGGTGGTGTCAT-3'	62	290	*Nco*I (2U)	290, 262, 55
**rs1800629 (*TNFA*)**	F5'-GGCAATAGGTTTTGAGGGCCAT-3'<br>R5'-TCCTCCCTGCTCCGATTCCG-3'	55	107	*Nco*I (2U)	107, 87, 20
**rs2227956 (*HSPA1L*)**	F5'-GGACAAGTCTGAGAAGGTACAG-3'<br>R5'-TAACTTAGATTCAGGTCTGG-3'	57	877	*NcoI* (2U)	877, 553, 324
**rs1061581 (*HSPA1B*)**	F5'-CATCGACTTCTACACGTCCA-3'<br>R5'-CAAAGTCCTTGAGTCCCAAC-3'	57	1146	*Pst*I (7U)	1146, 934, 183
**rs763780 (*IL17F*)**	F5'-GCACCAAGGCTGCTCTGTTTCTT-3'<br>R5'-GGTAAGGAGTGGCATTTCTACA-3'	55	145	*Nla*III (3U)	145, 86, 59
**rs4644 (*LGALS3*)**	F5'-CTCCATGATGCGTTATCTGGGTCTGG-3'<br>R5'-CAGTGGCCCAGCAGGGGCGCCATAGG-3'	57	324	*Nco*I (2U)	324, 171, 153
**rs1042522 (*TP53*)**	F5'-GAAGACCCAGGTCCAGATGA-3'<br>R5'-CTGCCCTGGTAGGTTTTCTG-3'	55	152	*BstU*I (2U)	152, 102, 50

**Polymorphisms genotyped by Real Time PCR using allelic discrimination TaqMan™ SNP Genotyping Assay**		
**Polymorphism (Gene)**	**Fluorescently labelled [VIC/FAM] MGB™ probes**	**TaqMan® assays ID**
**rs699947 (*VEGFA*)**	GCCAGCTGTAGGCCAGACCCTGGCA[A/C]GATCTGGGTGGATAATCAGACTGAC	C___8311602_10
**rs833061 (*VEGFA*)**	GAGTGTGTGCGTGTGGGGTTGAGGG[C/T]GTTGGAGCGGGGAGAAGGCCAGGGG	C___1647381_10
**rs2010963 (*VEGFA*)**	CGCGCGGGCGTGCGAGCAGCGAAAG[C/G]GACAGGGGCAAAGTGAGTGACCTGC	C___8311614_10
**rs3025039 (*VEGFA*)**	GCATTCCCGGGCGGGTGACCCAGCA[C/T]GGTCCCTCTTGGAATTGGATTCGCC	C___16198794_10
**rs689466 (*COX-2*)**	TTAGATGGAAGGGAGATTTTGACAG[C/T]TGGAATTTCATCTTTGCTTTTGTTT	C___2517145_20
**rs5275 (*COX-2*)**	TGTTTTTGTTTGATGACAGAAAAAT[A/G]ACCAAAAGTACTTTAAAATTTCAAA	C___7550203_10

**Polymorphisms genotyped by Real Time PCR using allelic discrimination custom TaqMan® assays**		
**Polymorphism (Gene)**	**Primer sequences and fluorescently labelled [VIC/FAM] MGB™ probes**	**Product size**
**rs6917 (*PHB*)**	F5'-TTGGTCCCTCTCAGATACCCA-3'<br>R5'-CCGTGAGAAGGGCAGTCTCT-3'<br>P5'-CTGCCAAAGA[T/C]GTGT-3'	131bp
p.R337H (***TP53***)	F5'-CCTCCTCTGTTGCTGCAGATC-3'<br>R5'-CCTCATTCAGCTCTCGGAACAT-3'<br>P5'-CGTGAGC[G/A]CTTCGAG-3'	64bp

PCR: Polymerase Chain Reaction; RFLP: Restriction Fragment Length Polymorphism; SNP: single nucleotide polymorphism; R: reverse; F: forward; P: probe; bp: base pair; ID: identification code for the validated TaqMan® SNP Genotyping Assays.

Table S2. General characteristics of the studied sample and comparison of the sociodemographic status, smoking and alcohol consumption between controls and cases (both considering the total sample and the cases stratified for the diffuse histological subtype).

	CONTROLS	CASES									
**Charactheristics**	**Total sample N (%)**	**Total sample N (%)**	**χ^2/U**	***p***	**OR (95% CI)**	***p*[#]**	**Diffuse subtype N (%)**	**χ^2/U**	***p***	**OR (95% CI)**	***p*[#]**
	**N=262**	**N=178**					**N=112**				
**Age**											
Median (IQR) years old	57 (26)	62 (21)	21155	0.098[a]	1.0 (1.00-1.03)	0.040*	60.5 (21)	14396	0.773[a]	1.01 (0.99-1.02)	0.53
**Gender**											
Female	143 (54.6)	69 (38.8)	10.6	0.001[b,*]	1 (Ref)		42 (37.5)	9.157	0.002[b,*]	1 (Ref)	
Male	119 (45.4)	109 (61.2)			1.9 (1.3-2.8)	<0.001*	70 (62.5)			2.0 (1.3-3.2)	0.003*
**Ethnicity**											
White	214 (82.3)	134 (75.3)	6.05	0.108[b]	1 (Ref)		84 (75.0)	4.508	0.212[b]	1 (Ref)	
Brown	25 (9.6)	25 (14.0)			1.6 (0.9-2.9)	0.12	16 (14.3)			1.63 (.8-3.2)	0.16
Black	18 (6.9)	12 (6.7)			1.1 (0.5-2.3)	0.87	8 (7.1)			1.13 (0.5-2.7)	0.78
Yellow	3 (1.2)	7 (3.9)			3.7 (0.9-14.6)	0.06	4 (3.6)			3.4 (0.7-15.5)	0.11
**Educational level**											
0 to 5 years	56 (23.5)	44 (26.2)	7.024	0.068[b]	1 (Ref)		28 (26.4)	6.119	0.106[b]	1 (Ref)	
6 to 9 years	124 (52.1)	103 (61.3)			1.0 (0.6-1.6)	0.96	66 (62.3)			1.02 (0.6-1.7)	0.96
10 to 12 years	42 (17.6)	14 (8.3)			0.6 (0.3-1.1)	0.10	8 (7.5)			0.51 (0.2-1.2)	0.13
> 12 years	16 (6.7)	7 (4.2)			0.3 (0.1-1.1)	0.07	4 (3.8)			0.27 (0.1-1.2)	0.09
**Smoking status**											
Never	161 (61.5)	66 (37.1)	26.169	<0.001[b,*]	1 (Ref)		40 (35.7)	24.112	<0.001[b,*]	1 (Ref)	
In the past	66 (25.2)	66 (37.1)			2.4 (1.6-23.8)	<0.001*	38 (33.9)			2.3 (1.4-3.9)	0.002*
Current	35 (13.4)	46 (25.8)			3.2 (1.9-5.4)	<0.001*	34 (30.4)			3.9 (2.2-7.0)	<0.001*
**Drinking status**											
Never	213 (81.3)	94 (52.8)	43.527	<0.001[b,*]	1 (Ref)		55 (49.1)	42.335	<0.001[b,*]	1 (Ref)	
In the past	20 (7.6)	47 (26.4)			5.3 (3.0-9.5)	<0.001*	31 (27.7)			6.0 (3.2-11.3)	<0.001*
Current	29 (11.1)	37 (20.8)			2.9 (17-5.0)	<0.001*	26 (23.2)			3.5 (1.9-6.4)	<0.001*

N: number of individuals; IQR: interquartile range; OR: Odds ratio; 95% CI: 95% Confidence Interval; Ref: reference; [a] Mann Whitney test (U); [b] Chi-Square test (χ^2); [#] Univariate Logistic Regression analysis; * p <0.05.

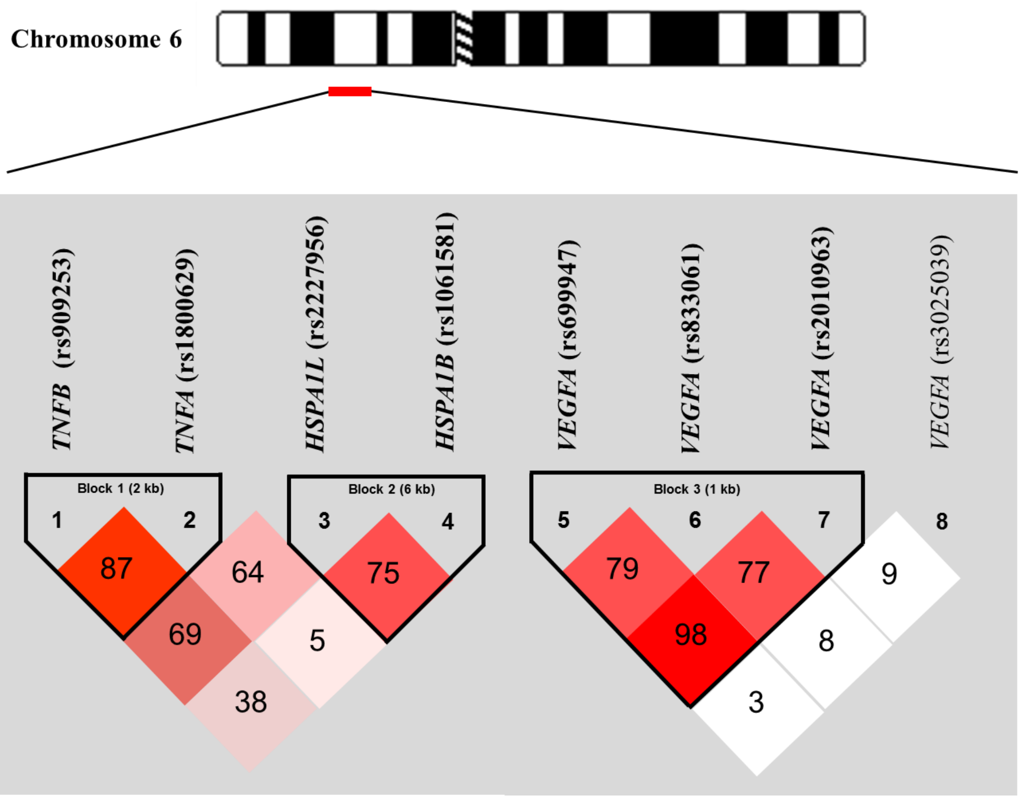

Figure S1. Representation of the haplotype blocks whose polymorphisms located on chromosome 6 (*TNFB*, *TNFA*, *HSP1AL*, *HSPA1B*, and *VEGFA* genes) were in linkage disequilibrium (LD) in the total sample of cases (N=178) and controls (N=262). Block 1 is composed by polymorphisms of the *TNFB*/*TNFA* genes; Block 2 by polymorphisms of *HSPA1L*/*HSPA1B* genes and Block 3 by polymorphisms of *VEGFA* gene. The numbers in squares indicate pairwise D' values and corresponding shade of red represents the degree of LD between the polymorphisms; LD considered when D' ≥ 0.75. Adapted from Haploview 4.2 software.

Table S3. Polymorphisms in linkage disequilibrium and haplotype association analyses with gastric cancer susceptibility.

Block	Polymorphisms (Genes)	Haplotypes	Frequency (%)	Cases/Controls (%)	χ^2	*p*
1	rs909253 and rs1800629 *(TNFB/TNFA)*	AG	64.4	64.3/64.4	0.00	0.98
		GG	21.9	22.5/21.5	0.11	0.74
		GA	12.6	11.6/13.4	0.60	0.44
		AA	1.1	1.6/0.7	1.49	0.22
2	rs2227956 and rs1061581 (*HSPA1L*/*HSPA1B*)	TG	54.2	57.0/52.2	1.89	0.17
		TA	38.0	36.3/39.3	0.80	0.37
		CA	6.7	5.2/7.8	2.13	0.14
		CG	1.1	1.5/0.7	1.24	0.27
3	rs699947, rs833061 and rs2010963 (*VEGFA*)	ACG	32.8	33.3/32.4	0.08	0.78
		CTC	32.4	37.6/28.6	7.66	0.006*
		CTG	23.3	24.7/22.3	0.69	0.40
		ATG	4.7	0.3/8.0	26.51	<0.001*
		CCG	3.4	3.1/3.7	0.21	0.65
		CCC	3.2	0.6/5.1	13.11	<0.001*

Haplotypes with frequency less than 1% were excluded of the analysis; * p <0.05. Haploview 4.2 software.

Table S4. Clinicopathological characteristics of the cases with gastric cancer at the time of diagnosis in the total sample and stratified by Lauren´s histological subtypes and results of the comparison of these parameters between Diffuse and Intestinal subtypes.

Clinicopathological characteristics	Cases N (%)			χ^2/U	*p*	OR (95% CI)[a]	*p*
	**Total cases N=178**	**Diffuse subtype N=112**	**Intestinal subtype N=59**				
**Age at diagnosis**							
Median (IQR) years old	62 (21)	60.5 (21)	66 (17)	2571.5	0.017[a],*	0.97 (0.95-0.99)	0.026*
**Gender**							
Female	143 (54.6)	42 (37.5)	24 (41.7)	0.17	0.685[b]	1 (Ref)	
Male	119 (45.4)	70 (62.5)	35 (59.3)			1.1 (0.6-2.2)	0.69
**Histological subtype**							
Intestinal	59 (33.1)	-	59 (100.0)	-	-	-	-
Diffuse	112 (62.9)	112 (100.0)	-	-	-	-	-
Mixed	7 (3.9)	-	-	-	-	-	-
**Tumor size**							
≤ 5 cm	94 (52.8)	61 (54.5)	33 (55.9)	0.03	0.854[b]	1 (Ref)	
> 5 cm	84 (47.2)	51 (45.4)	26 (44.1)			1.1 (0.6-2.0)	0.854
**Perineural invasion**							
No	76 (44.2)	38 (34.9)	34 (60.7)	10.1	0.002[b,*]	1 (Ref)	
Yes	96 (55.8)	71 (65.1)	22 (39.3)			2.9 (1.5-5.6)	0.002*
**Lymphatic invasion**							
No	68 (39.5)	38 (34.5)	27 (49.1)	3.2	0.071[b]	1 (Ref)	
Yes	104 (60.5)	72 (65.5)	28 (50.9)			1.8 (0.9-3.5)	0,073
**Vascular invasion**							
No	81 (49.4)	44 (43.6)	33 (58.9)	3.4	0.065[b]	1 (Ref)	
Yes	83 (50.6)	57 (56.4)	23 (41.1)			1.9 (0.9-3.6)	0.066
**Inflammatory infiltration**							
none to weak	50 (49.5)	34 (53.1)	15 (50.0)	0.08	0.777[b]	1 (Ref)	
moderate to intense	51 (50.5)	30 (46.9)	16 (50.0)			0.9 (0.4-2.1)	0.777
**Desmoplasia**							
none to weak	39 (37.9)	18 (26.9)	17 (58.6)	8.8	0.003[b,*]	1 (Ref)	
moderate to intense	64 (62.1)	49 (73.1)	12 (41.4)			3.9 (1.5-9.6)	0.004*
**Depth of invasion (pT)**							
t1+t2	36 (20.3)	20 (18.0)	16 (27.1)	1.9	0.167[b]	1 (Ref)	
t3+t4	141 (79.7)	91 (82.0)	43 (72.9)			1.7 (0.8-3.6)	0.169
**Lymph nodes metastasis**							
No	40 (22.6)	17 (15.3)	23 (39.0)	12.0	0.001[b,*]	1 (Ref)	
Yes	137 (77.4)	94 (84.7)	36 (61.0)			3.5 (1.7-7.4)	0.001*
**Distant metastasis (pM)**							
No	152 (85.4)	94 (83.9)	52 (88.1)	0.55	0.459[b]	1 (Ref)	
Yes	26 (14.6)	18 916.1)	7 (11.9)			1.4 (0.6-3.6)	0.461
**TNM staging**							
I+II	54 (30.5)	27 (24.3)	26 (44.1)	7.0	0.008[b,*]	1 (Ref)	
III+IV	123 (69.5)	84 (75.7)	33 (55.9)			2.5 (1.3-4.8)	0.009*

N: number of individuals; IQR: interquartile range; TNM based on the 7th edition of UICC/AJCC, 2010; [a] Mann Whitney test (U); [b] Chi-Square test (χ^2); OR: Odds Ratio; 95% CI: 95% Confidence Interval; Ref: reference; [a] OR calculation was based on Diffuse in relation to Intestinal subtype; * p <0.05.

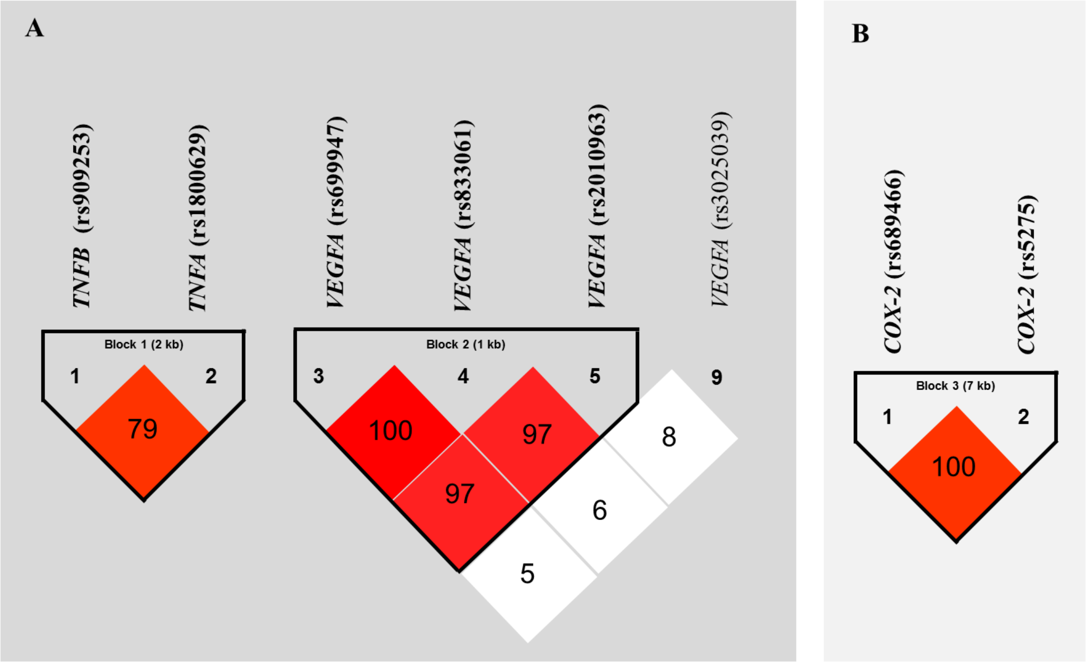

Figure S2. Representation of the haplotype blocks whose polymorphisms were found in linkage disequilibrium (LD) in the subgroup of cases with gastric cancer. (A) Block 1 is composed by polymorphisms of the *TNFB*/*TNFA* genes; Block 2 by polymorphisms of *VEGFA* gene and (B) Block 3 by polymorphisms of *COX-2* gene. The numbers in squares indicate pairwise D' values and corresponding shade of red represents the degree of LD between the polymorphisms; LD considered when D' ≥ 0.75. Adapted from Haploview 4.2 software.

Table S5. Polymorphisms in linkage disequilibrium in the sample of cases (N=178) and haplotype association analyses with anatomopathological features of gastric cancer patients.

**Block**	**Polymorphisms (Genes)**	**Haplotypes**	**freq (%)**	**Anatomopathological characteristics**	**Presence/Absence (%)**	χ^2	***p***
1	rs909253 and rs1800629 (*TNFB/TNFA*)	AG	64.2	-	-	-	-
		GG	22.6	Perineural invasion	26.0/17.0	4.05	0.044*
		GA	11.4	-	-	-	-
		AA	1.8	-	-	-	-
2	rs699947, rs833061 and rs2010963 (*VEGFA*)	CTC	38.2	-	-	-	-
		ACG	33.6	Vascular invasion	39.8/26.7	6.26	0.012*
		CTG	24.5	-	-	-	-
		CCG	3.4	-	-	-	-
		CCC	3.2	-	-	-	-
3	rs689466 and rs5275 (*COX-2*)	AT	45.2	-	-	-	-
		AC	37.4	-	-	-	-
		GT	17.4	Intestinal histological subtype	23.7/14.7	4.27	0.038*

freq: haplotype frequency in the sample of case individuals; * p <0.05. Haploview 4.2 and PLINK softwares.

Table S6. Overall and Disease-free survival by anatomopathological features, stratified for the cases with the diffuse histological subtype (N=112).

Anatomopathological characteristics	Categories	Overall Survival						Disease-free Survival					
		**Cases N**	**Events N**	**Mean**	**log-rank *p***	**HR (95% CI)**	***p***	**Cases N**	**Events N**	**Mean**	**log-rank *p***	**HR (95 % CI)**	***p***
Tumor size	≤ 5 cm	61	35	82.4	0.032*	1 (Ref)	0.034*	61	25	100.7	0.014*	1 (Ref)	0.016*
	> 5 cm	51	39	44.2		1.7 (1.0-2.6)		51	34	50.3		1.9 (1.1-3.2)	
Depth of invasion (pT)	t1+t2	20	10	99.1	0.036*	1 (Ref)	0.040*	20	5	128.8	0.006*	1 (Ref)	0.010*
	t3+t4	91	63	61.1		2.0 (1.0-3.9)		91	53	70.2		3.3 (1.3-8.4)	
Perineural invasion	no	38	21	88.8	0.019*	1 (Ref)	0.021*	38	18	96.0	0.120	1 (Ref)	0.123
	yes	71	51	56.2		1.8 (1.1-3.0)		71	39	71.5		1.6 (0.9-1.7)	
Lymphatic invasion	no	38	18	104.0	<0.001*	1 (Ref)	<0.001*	38	11	125.9	<0.001*	1 (Ref)	<0.001*
	yes	72	54	46.3		2.7 (1.6-4.6)		72	46	53.7		3.9 (2.0-7.5)	
Vascular invasion	no	44	23	95.8	0.001*	1 (Ref)	0.001*	44	16	113.9	<0.001*	1 (Ref)	<0.001*
	yes	57	42	45.7		2.3 (1.4-3.9)		57	36	53.3		2.9 (1.6-5.3)	
Inflammatory infiltration	none to weak	34	22	72.9	0.820	1 (Ref)	0.820	34	20	77.6	0.734	1 (Ref)	0.734
	moderate to strong	30	19	74.4		1.1 (0.6-2.0)		30	15	89.3		0.9 (0.5-1.7)	
Desmoplasia	none to weak	18	11	78.4	0.495	1 (Ref)	0.496	18	11	79.2	0.947	1 (Ref)	0.947
	moderate to strong	49	33	68.6		1.3 (0.6-2.5)		49	27	80.6		1.0 (0.5-2.1)	
Lymph nodes metastasis	no	17	7	110.8	0.022*	1 (Ref)	0.027*	17	3	139.0	0.007*	1 (Ref)	0.013*
	yes	94	66	59.2		2.4 (1.1-5.3)		94	55	70.9		4.3 (1.4-13.9)	
Distant metastasis (pM)	no	94	56	78.4	<0.001*	1 (Ref)	<0.001*	94	41	95.7	<0.001*	1 (Ref)	<0.001*
	yes	18	18	17.4		3.4 (1.9-5.8)		18	18	16.4		4.0 (2.3-7.0)	
TNM staging	I+II	27	11	111.4	0.001*	1 (Ref)	0.002*	27	4	146.0	<0.001*	1 (Ref)	<0.001*
	III+IV	84	62	52.7		2.8 (1.4-5.3)		84	54	60.5		6.4 (2.3-17.8)	

N: number of individuals; Mean: mean survival time in months; HR: Hazard Ratio; 95% CI: 95% Confidence Interval; Ref: reference; * p <0.05.

Table S7. Overall and Disease-free survival by anatomopathological features in the total sample of gastric cancer patients (N=178).

Anatomopathological characteristics	Categories	Overall Survival						Disease-free Survival					
		Cases N	Events N	Mean	log-rank *p*	HR (95% CI)	*p*	Cases N	Events N	Mean	log-rank *p*	HR (95 % CI)	*p*
Tumor size	≤ 5 cm	94	46	95.13	0.017*	1.0 (Ref)	0.018*	94	31	115.3	0.001*	1.0 (Ref)	<0.001*
	> 5 cm	84	53	59.85		1.6 (1.1-2.4)		84	48	64.2		2.8 (1.7-4.6)	
Depth of invasion (pT)	t1+t2	36	14	112.29	0.007*	1.0 (Ref)	0.008*	36	7	137.3	0.001*	1.0 (Ref)	0.003*
	t3+t4	141	84	74.00		2.1 (1.2-3.8)		141	71	83.2		3.6 (1.5-8.3)	
Perineural invasion	no	76	32	104.39	<0.001*	1.0 (Ref)	0.001*	76	27	111.7	0.006*	1.0 (Ref)	0.006*
	yes	96	64	63.40		2.1 (1.4-3.2)		96	50	78.3		2.0 (1.2-3.3)	
Lymphatic invasion	no	68	27	110.18	<0.001*	1.0 (Ref)	<0.001*	68	17	129.7	<0.001*	1.0 (Ref)	<0.001*
	yes	104	69	58.13		2.4 (1.5-3.8)		104	60	64.7		2.9 (1.6-5.0)	
Vascular invasion	no	81	32	109.49	<0.001*	1.0 (Ref)	<0.001*	81	24	121.8	<0.001*	1.0 (Ref)	<0.001*
	yes	83	56	56.39		2.4 (1.6-3.8)		83	47	66.5		3.0 (1.7-5.3)	
Inflammatory infiltration	none to weak	50	30	75.07	0.376	1.0 (Ref)	0.378	50	26	83.3	0.400	1.0 (Ref)	0.296
	moderate to strong	51	26	92.52		0.9 (4.7-1.3)		51	22	100.4		0.7 (0.4-1.3)	
Desmoplasia	none to weak	39	19	92.36	0.238	1.0 (Ref)	0.240	39	18	94.1	0.454	1.0 (Ref)	0.776
	moderate to strong	64	39	77.25		1.4 (0.8-2.4)		64	33	86.4		1.1 (0.6-2.1)	
Lymph nodes metastasis	no	40	14	118.42	0.002*	1.0 (Ref)	0.003*	40	4	153.7	<0.001*	1.0 (Ref)	<0.001*
	yes	137	84	70.25		2.4 (1.3-4.2)		137	74	78.0		6.1 (2.2-17.0)	
Distant metastasis (pM)	no	152	76	92.19	<0.001*	1.0 (Ref)	<0.001*	152	56	107.8	<0.001*	1.0 (Ref)	<0.001*
	yes	26	23	19.39		3.9 (2.4-6.3)		26	23	18.5		5.0 (2.8-9.0)	
TNM staging	I+II	54	18	120.59	<0.001*	1.0 (Ref)	<0.001*	54	7	149.1	<0.001*	1.0 (Ref)	<0.001*
	III+IV	123	80	63.08		2.8 (1.7-4.7)		123	71	69.8		5.9 (2.7-13.1)	
Lauren´s histological classification	Intestinal	59	23	99.97	0.003*	1.0 (Ref)	0.005*	59	18	109.6	0.008*	1.0 (Ref)	0.009*
	Diffuse	112	74	68.16		2.0 (1.3-3.2)		112	59	81.1		2.0 (1.2-3.4)	

N: number of individuals; Mean: mean survival time in months; HR: Hazard Ratio; 95% CI: 95% Confidence Interval; Ref: reference; * $p < 0.05$.

Table S8. In silico prediction for the functional effect in the final coded protein for the studied polymorphisms that lead to amino acid change.

Polymorphism (Gene)	Chromosome	Amino acid change	Polyphen2	SIFT
rs1052133 (*OGG1*)	3	Ser326Cis	benign	tolerated
rs2227956 (*HSPA1L*)	6	Thr493Met	benign	tolerated
rs763780 (*IL17F*)	6	His161Arg	benign	tolerated
rs4644 (*LGALS3*)	14	Pro64His	possibly pathogenic	deleterious
rs1042522 (*TP53*)	17	Arg72Pro	benign	tolerated
p.R337H (*TP53*)[a]	17	Arg337His	possibly pathogenic	deleterious

[a] genetic variation described as mutation; Polyphen-2: Polymorphism Phenotyping v2; SIFT: Sorting Intolerant From Tolerant.

Text S1: Discussion of the polymorphisms that did not present any relevant association in our study.

Here we discuss the selected polymorphisms included in our study that did not present any relevant association.

Among the four studied *VEGFA* polymorphisms, we did not find any association regarding the rs2010963 polymorphism. A previous study in breast cancer showed that this SNP was associated with several factors related to a worse progression and higher aggressiveness of the disease (increased susceptibility risk, higher *VEGFA* mRNA levels, tumor with bigger sizes, presence of perineural invasion, higher staging and shorter disease-free survival) [1].

Another cytokine that was selected for investigation in our study was IL17F. The rs763780 (*IL17F*) polymorphism was associated with gastric cancer in the Allele Model, but this significance was lost in the multivariate analysis. *IL17F* is part of a gene family with important involvement in tissue inflammation by inducing the expression of several other cytokines and chemokines. This polymorphism leads to a His to Arg substitution at amino acid 161 and *in vitro* analysis showed that the polymorphic variant loses the ability to activate the production of the mitogen-activated protein kinase pathway and certain cytokines and chemokines [2]. Our in silico analysis showed that this amino acid change is tolerated/benign for the final coded product. Although this SNP has been previously associated with risk and progression of gastric cancer [3], we did not find associations to any clinicopathological variable or prognosis in our study. The frequency of the polymorphic allele was too low (8.4% and 4.8% in cases and controls, respectively) and it also presented deviation from HWE in our sample. Therefore, this result should be reanalyzed in an independent and increased set of sample. We cannot exclude the hypothesis that this SNP has an impact on gastric cancer risk and progression because its function might be compensated by other redundant molecules inside the same pathway.

Regarding the rs1061581 (*HSPA1B*) polymorphism, we did not find any association neither with susceptibility, progression nor prognosis in our sample. It causes a silent substitution and has been described as able to regulate the protein expression interfering with its secondary structure and mRNA stability, affecting its anti-apoptotic effect and its function as a modulator of the immune system [4,5].

OGG1 is a DNA glycosylase that belongs to the BER (Base Excision Repair) pathway, which repairs mainly endogenous/oxidative lesions as result of the cellular metabolism [6]. We hypothesized that maybe functional polymorphisms in repair genes could influence in the capacity of the organism repair DNA damage caused by diet carcinogens, oxidative stress or inflammation induced by *H. pylori*, in the gastric mucosa. Also, OGG1 has been shown to be important as a modulator of the immune and inflammatory systems [7]. We selected the rs1052133 polymorphism for investigation, which is located in the exon 7 and results in a change from Ser to Cis in 326 position of the protein. The in silico analysis by Polyphen and SIFT softwares showed that this change is tolerated/benign. However, Cis variant has demonstrated to increase the genetic instability and decrease the repair rate of 8-oxoguanine in vivo [8]. Nevertheless, no association was found with this polymorphism in the present study.

Although some studies have described a functional role for the rs6917 polymorphism, located in the 3′ UTR of the *PHB* gene [9] and that it has been associated with an increased risk for development of some types of tumors [10,11], in the present study we did not find association with gastric cancer susceptibility. No association was found regarding progression and prognosis as well. Our group has been studying the role of PHB and we have observed that the TT genotype increased the risk for melanoma in the presence of specific host risk factor [12]. Another study from our group demonstrated a possible role for this polymorphism in the transcriptional regulation of PHB in gastric cancer once T allele was associated with reduced *PHB* expression levels [13]. Therefore, functional studies on prohibitin polymorphism are necessary to elucidate its functional role.

References

1. Sa-Nguanraksa, D.; Chuangsuwanich, T.; Pongpruttipan, T.; Kummalue, T.; Rojananin, S.; Ratanawichhitrasin, A.; Prasarttong-Osoth, P.; Chuthatisith, S.; Pisarnturakit, P.; Aeumrithaicharoenchok, W., et al. Vascular endothelial growth factor -634G/C polymorphism is associated with increased breast cancer risk and aggressiveness. *Mol Med Rep* **2013**, *8*, 1242-1250, doi:10.3892/mmr.2013.1607.
2. Kawaguchi, M.; Takahashi, D.; Hizawa, N.; Suzuki, S.; Matsukura, S.; Kokubu, F.; Maeda, Y.; Fukui, Y.; Konno, S.; Huang, S.K., et al. IL-17F sequence variant (His161Arg) is associated with protection against asthma and antagonizes wild-type IL-17F activity. *J Allergy Clin Immunol* **2006**, *117*, 795-801, doi:10.1016/j.jaci.2005.12.1346.

3. Wu, X.; Zeng, Z.; Chen, B.; Yu, J.; Xue, L.; Hao, Y.; Chen, M.; Sung, J.J.; Hu, P. Association between polymorphisms in interleukin-17A and interleukin-17F genes and risks of gastric cancer. *Int J Cancer* **2010**, *127*, 86-92, doi:10.1002/ijc.25027.
4. Schroeder, S.; Bischoff, J.; Lehmann, L.E.; Hering, R.; von Spiegel, T.; Putensen, C.; Hoeft, A.; Stüber, F. Endotoxin inhibits heat shock protein 70 (HSP70) expression in peripheral blood mononuclear cells of patients with severe sepsis. *Intensive Care Med* **1999**, *25*, 52-57.
5. Wu, Y.R.; Wang, C.K.; Chen, C.M.; Hsu, Y.; Lin, S.J.; Lin, Y.Y.; Fung, H.C.; Chang, K.H.; Lee-Chen, G.J. Analysis of heat-shock protein 70 gene polymorphisms and the risk of Parkinson's disease. *Hum Genet* **2004**, *114*, 236-241, doi:10.1007/s00439-003-1050-1.
6. Palli, D.; Polidoro, S.; D'Errico, M.; Saieva, C.; Guarrera, S.; Calcagnile, A.S.; Sera, F.; Allione, A.; Gemma, S.; Zanna, I., et al. Polymorphic DNA repair and metabolic genes: a multigenic study on gastric cancer. *Mutagenesis* **2010**, *25*, 569-575, doi:10.1093/mutage/geq042.
7. Mabley, J.G.; Pacher, P.; Deb, A.; Wallace, R.; Elder, R.H.; Szabó, C. Potential role for 8-oxoguanine DNA glycosylase in regulating inflammation. *FASEB J* **2005**, *19*, 290-292, doi:10.1096/fj.04-2278fje.
8. Bravard, A.; Vacher, M.; Moritz, E.; Vaslin, L.; Hall, J.; Epe, B.; Radicella, J.P. Oxidation status of human OGG1-S326C polymorphic variant determines cellular DNA repair capacity. *Cancer Res* **2009**, *69*, 3642-3649, doi:10.1158/0008-5472.CAN-08-3943.
9. Manjeshwar, S.; Branam, D.E.; Lerner, M.R.; Brackett, D.J.; Jupe, E.R. Tumor suppression by the prohibitin gene 3'untranslated region RNA in human breast cancer. *Cancer Res* **2003**, *63*, 5251-5256.
10. Jupe, E.R.; Badgett, A.A.; Neas, B.R.; Craft, M.A.; Mitchell, D.S.; Resta, R.; Mulvihill, J.J.; Aston, C.E.; Thompson, L.F. Single nucleotide polymorphism in prohibitin 3' untranslated region and breast-cancer susceptibility. *Lancet* **2001**, *357*, 1588-1589.
11. Zhou, T.B.; Yin, S.S.; Huang, J.J.; Ou, C. Relationship between the prohibitin 3' untranslated region C > T gene polymorphism and cancer susceptibility--results of a meta-analysis. *Asian Pac J Cancer Prev* **2012**, *13*, 3319-3323.
12. Francisco, G.; Gonçalves, F.T.; Luiz, O.C.; Saito, R.F.; Toledo, R.A.; Sekiya, T.; Tortelli, T.C.; Violla, E.D.; Furuya Mazzotti, T.K.; Cirilo, P.D., et al. Polymorphisms in the p27kip-1 and prohibitin genes denote novel genes associated with melanoma risk in Brazil, a high ultraviolet index region. *Melanoma Res* **2013**, *23*, 231-236, doi:10.1097/CMR.0b013e3283612483.
13. Leal, M.F.; Cirilo, P.D.; Mazzotti, T.K.; Calcagno, D.Q.; Wisnieski, F.; Demachki, S.; Martinez, M.C.; Assumpção, P.P.; Chammas, R.; Burbano, R.R., et al. Prohibitin expression deregulation in gastric cancer is associated with the 3' untranslated region 1630 C>T polymorphism and copy number variation. *PLoS One* **2014**, *9*, e98583, doi:10.1371/journal.pone.0098583.
